# Evolutionary chemical space exploration for functional materials: computational organic semiconductor discovery†

**DOI:** 10.1039/d0sc00554a

**Published:** 2020-04-22

**Authors:** Chi Y. Cheng, Josh E. Campbell, Graeme M. Day

**Affiliations:** Computational Systems Chemistry, School of Chemistry, University of Southampton Highfield Southampton SO17 1NX UK g.m.day@soton.ac.uk

## Abstract

Computational methods, including crystal structure and property prediction, have the potential to accelerate the materials discovery process by enabling structure prediction and screening of possible molecular building blocks prior to their synthesis. However, the discovery of new functional molecular materials is still limited by the need to identify promising molecules from a vast chemical space. We describe an evolutionary method which explores a user specified region of chemical space to identify promising molecules, which are subsequently evaluated using crystal structure prediction. We demonstrate the methods for the exploration of aza-substituted pentacenes with the aim of finding small molecule organic semiconductors with high charge carrier mobilities, where the space of possible substitution patterns is too large to exhaustively search using a high throughput approach. The method efficiently explores this large space, typically requiring calculations on only ∼1% of molecules during a search. The results reveal two promising structural motifs: aza-substituted naphtho[1,2-*a*]anthracenes with reorganisation energies as low as pentacene and a series of pyridazine-based molecules having both low reorganisation energies and high electron affinities.

## Abstract 2

Evolutionary optimisation and crystal structure prediction are used to explore chemical space for molecular organic semiconductors.

## Graphical abstract

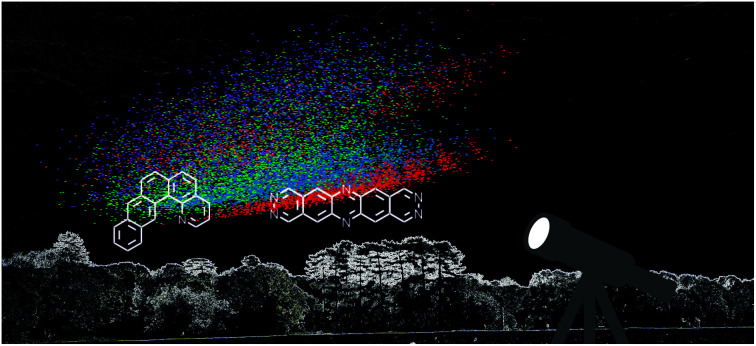

## 1 Introduction

The field of crystal engineering aims to design new materials with targeted properties from an understanding of how intermolecular interactions govern their crystal structures. The field has mainly been developed empirically, through systematic studies of observed crystal structures, enabled by their collection in crystallographic databases.^1,2^ A complementary approach is *ab initio* crystal structure prediction (CSP), based on exploring the crystal packing space available to a molecule.^3,4^

Once promising molecules have been identified, either by chemical intuition or other methods, the CSP approach can be a powerful tool, especially when combined with property prediction of the predicted crystal structures. The result is an energy-structure–function (ESF) map for each molecule, describing the likely crystal structures, their energetic stability and properties.^5^ As an example of their utility, ESF maps have guided the discovery of a set of unprecedentedly low density molecular crystals with high methane storage capacity.^6^ In the field of organic semiconductors, ESF maps have been used to investigate the effect of crystal packing types on calculated carrier mobility within families of azapentacene and pyrrole-based azaphenacene molecules.^7,8^ Others have used ESF maps to understand the influence of chiral composition on carrier mobilities for the predicted crystal structures of a [6]helicene molecule.^9^

One of the major limitations for the use of computational screening in functional materials discovery is the need to identify which molecules to study from the vast chemical space of possible targets. A high-throughput approach is restricted by the relatively high computational cost of CSP compared to single molecule calculations; CSP is currently usually applied to the detailed study of a single molecule, and occasionally to relatively small families of molecules. One strategy that avoids the need for CSP, which has been applied successfully to identify a high carrier mobility organic crystal,^10^ is to assess molecules using the assumption that their crystal packing will be analogous to known, related experimental structures. The risk with this approach is to miss new materials whose promising properties result from an unexpected crystal packing motif. An alternative approach is to screen crystallographic databases of known materials,^11^ which can be particularly efficient because the crystal structures are known and the targeted molecules are likely to be commercially available or synthetically accessible.

The goal of the present work is the implementation of an evolutionary framework for exploration of chemical space to be used to feed into a CSP process for molecular evaluation. Our vision is that, instead of deciding on a single molecule or small family of molecules for investigation through synthesis, crystallisation and characterisation, the researcher must only decide on a broadly defined region of chemical space and uses computational methods to identify the most promising candidate molecules. We therefore occupy an intermediate searching regime between the high throughput database processing of candidates and molecular design by chemical intuition methods. This approach has the advantage of discovering new molecules and crystal structures not included within a given database, searching through many more molecules than chemical design strategies, whilst maintaining some aspects of chemical intuition through the specification of chemical space. We illustrate the method by targeting the discovery of small molecule organic semiconductors with high electron mobilities.

One crucial property for organic semiconductors is the reorganisation energy, which determines the activation barrier for carrier hopping between sites in hopping models of charge transport and should be minimised to increase carrier mobility in a molecular semiconductor.^12^ Carrier mobilities can also be improved by optimising π–π stacking between molecular units, leading to larger intermolecular electronic coupling and higher charge carrier hopping rates. For organic field-effect transistor (OFET) devices, a Schottky barrier^13,14^ for carrier injection exists at the metal–semiconductor interface, due to a mismatch between the Fermi level of the electrode and conduction (for electron injection) or valence (for hole injection) band edge of the semiconductor. A decrease in this barrier corresponds to an increase in the injected charge current density from the metal to the semiconductor and therefore an overall increase in the efficiency of the OFET. The Schottky barrier therefore controls the n-type, p-type or ambipolar behavior of an OFET device, depending on the height of the Schottky barrier for electron or hole injection.^13^

Therefore, to find the optimum organic semiconductor material for an n-type OFET device requires the maximisation of the electronic couplings and minimisation of both the reorganisation energies and Schottky barriers for electron transport. These are all dependent on the crystal structures of the semiconductor, but both reorganisation energy and the Schottky barrier can be estimated from properties of the isolated molecule. In the initial, evolutionary optimisation stage we focus on optimising molecular properties from isolated molecule calculations. The best performing molecules from an evolutionary optimisation are passed to a second stage of evaluation, where CSP and electronic coupling calculations are used to generate ESF maps of electron mobilities, from which we identify the most promising molecules.

We restrict this initial study to a chemical space containing aza-substituted pentacenes and related polyaromatic hydrocarbons (PAHs). Nitrogen substitution has been proposed as an effective means of modifying the electronic properties of molecules,^15^ as well as influencing the crystal packing of PAHs through the formation of polar intermolecular interactions.^7^

## 2 Methods

**Fig. 1:**
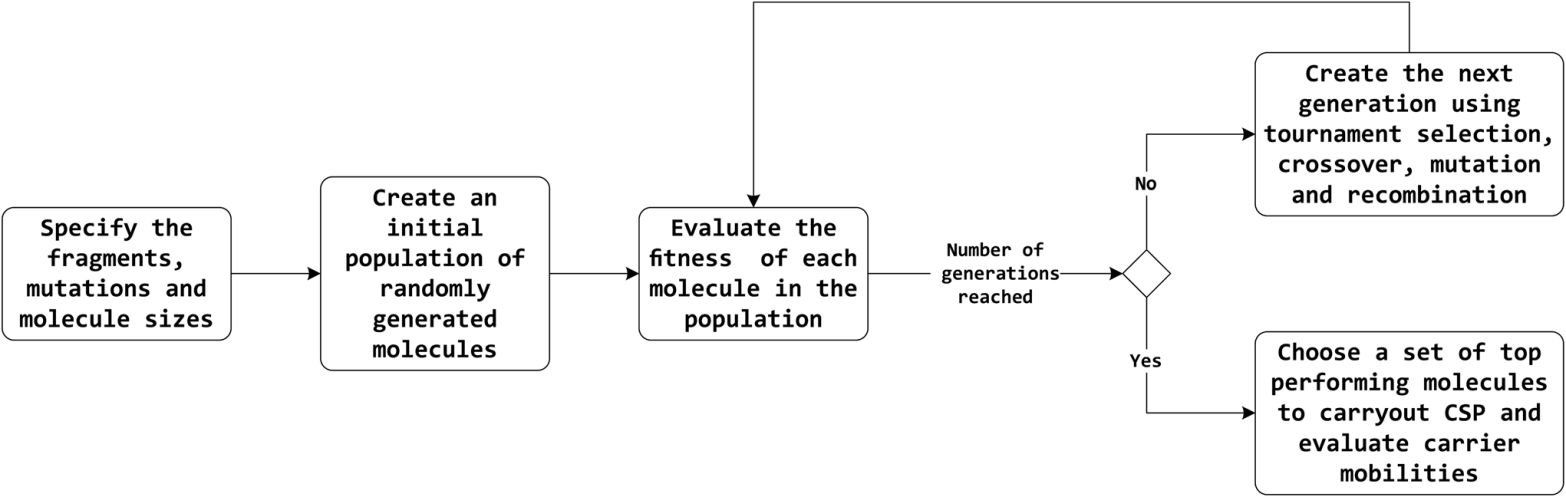
High-level flow diagram of the evolutionary algorithm optimisation process.

The overall workflow is outlined in Fig. 1. A brief description of methods is provided here, with full details in the ESI.†

### 2.1 Evolutionary search algorithm

A flexible evolutionary search algorithm was developed for the global optimisation of a molecule's chemical structure for a given calculated fitness function. The region of chemical space to be searched by the algorithm and the possible moves that can be made across chemical space are defined by three input variables and four transformation operations.

The three input variables—smiles, smarts and molsize—define molecular fragments that can be used by the algorithm to build or modify molecules. Smiles contains a list of SMILES strings^16,17^ representing molecules or fragments, acting as the primary building blocks for the creation of larger molecules. Smarts is a list of SMARTS strings^18,19^ which are used for fragment matching and mutations. Molsize defines the limits on the size of molecules that can be created where, for this work, we define size by the number of rings contained in a molecule.

**Fig. 2:**
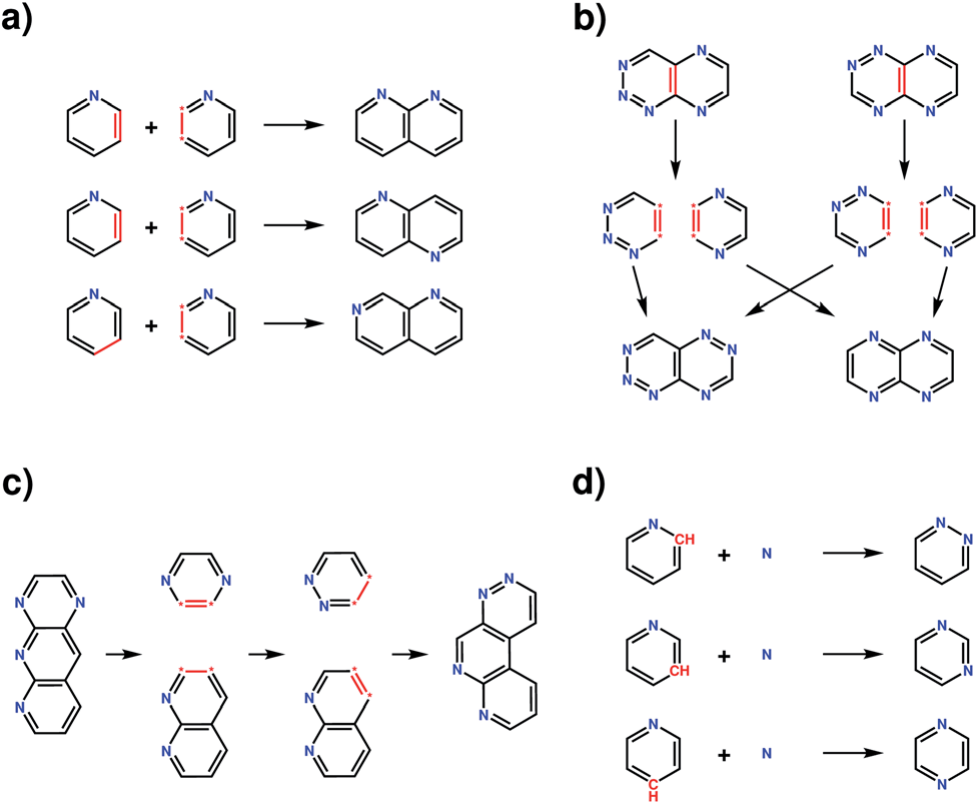
Examples of the four transformation operations implemented in the evolutionary algorithm. (a) The addition operation, illustrated with three possibilities for the addition of a pyridine fragment to a pyridine molecule, forming a naphthyridine type molecule; (b) crossover between two aza-napthalene molecules showing one crossover possibility for the example parent molecules. Additional possibilities can occur due to freedoms in the orientation of the fragments when combined together and the possible pairings of each fragment. (c) The recombination transformation of an aza-anthracene molecule, creating an isomer of the initial molecule. Additional possibilities can occur due to freedom in the fragmentation positions, fragmentation position moves and the orientation of the fragments when combined together. (d) The mutation transformation on the pyridine molecule with a nitrogen atom fragment, showing three possible mutations forming either a pyridazine, pyrimidine or pyrazine molecule.

The four transformation operations—addition, crossover, recombination and mutation—act by modifying one or more molecules (Fig. 2). Addition transforms a molecule into a larger molecule by the attachment of a new fragment, by first randomly selecting a possible bonding position, then orientation, for attachment. The molecule and fragment are then added together to create a larger molecule (Fig. 2a). Crossover fragments two parent molecules, each into two parts at a random position. Two child molecules are generated by combining two fragments (one from each parent) together (Fig. 2b). Recombination fragments a single molecule at a random position. The fragments are recombined after moving the fragmented positions, generating an isomer of the initial molecule (Fig. 2c). In mutation, a position on the molecule that is matched by any SMARTS string from the smarts list variable is randomly selected and replaced by a different fragment randomly selected from the same list (Fig. 2d). In this work, the addition and mutation operations were used for the generation of an initial population whilst crossover, recombination and mutation were used for the generation of new populations.

The initial population consisted of 100 randomly generated molecules for each run of the evolutionary algorithm using the input variables and transformation operations. Each molecule was created by randomly selecting one of the base molecules from the smiles list, to which the addition operation was applied using a second, randomly selected fragment from the same list. Further applications of the addition operation with further fragments were carried out until a randomly selected size within the limits given by molsize was reached. In this study, we have restricted the minimum and maximum sizes to be 5. A large number (500) of mutation operations using the smarts variable were then applied to the molecule.

The fitness of each molecule in the population was evaluated based on its calculated properties (see below). New generations of molecules were created using an elitism rate of 10%: the new population is made from the top 10% best performing molecules from the previous population. The remaining 90% is made using child molecules created based on crossover between parent molecules selected by 2-way tournament selections. Each child molecule then has a probability of 5% to undergo mutation and a probability of 5% to undergo recombination.

Newer generations are created continually until a desired number of generations or the convergence criteria are reached. Here, we ran all searches for a total of 30 generations. Since the selection and replication for the creation of new molecules in the next generation favour fitter molecules, the search algorithm is driven to a global minimum or maximum.

### 2.2 Chemical search space

**Fig. 3:**
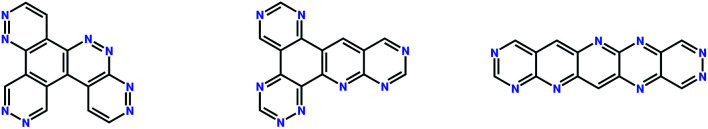
Chemical diagrams of three randomly generated molecules from the chemical space considered in this study.

In this study, we explore the region of chemical space of all aza-substituted isomers of pentacene, allowing any number of nitrogen atom substitutions and all connectivities of five 6-membered aromatic rings. The exception is that, in this work, addition of fragments to cove, bay and fjord regions^20^ was not allowed, so that the formation of pyrene-like ring arrangements is excluded. The total chemical space searched was determined to contain 68 064 unique molecules. Three randomly generated molecules from this space are shown in Fig. 3.

### 2.3 Fitness function

The evolutionary search algorithm was run ten times for each of two different fitness functions. The first fitness function,

1 *F*_A_ = *λ*_−_

− where *λ*_−_ is the reorganisation energy for electron transport calculated for the isolated molecule – was used to search for molecules with the best likelihood of forming crystal structures with high electron mobilities. The reorganisation energy *λ*_−_ for electron transport between two molecules was approximated using the four-point scheme using isolated molecule energies.^12^

2 *λ*_−_ = [*E*_−_(*R*_0_) − *E*_0_(*R*_0_)] + [*E*_0_(*R*_−_) − *E*_−_(*R*_−_)]

*E*_−_ and *E*_0_ are the energies of the anion and neutral molecules, respectively, calculated at the optimised geometries of the anion (*R*_−_) and neutral (*R*_0_) molecule. We also used *F*_A_ to evaluate the performance and reproducibility of the evolutionary algorithm.

The second fitness function,3
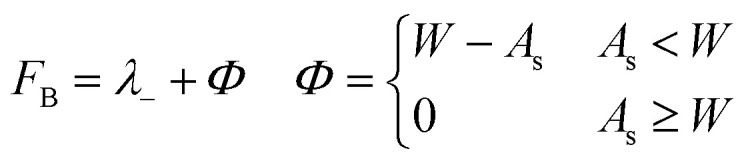
is a simple attempt at multi-objective optimisation, aiming to minimise both the barrier for injection of an electron into the semiconductor and the barrier for hopping across the semiconductor in hopping transport models. The penalty function added to *λ*_−_ corresponds to the Schottky barrier (*Φ* = *W* − *A*_s_) from the Schottky–Mott rule for the injection of an electron from an electrode with a work function *W* into the semiconductor material with a solid-state electron affinity *A*_s_,^13,14^ but is only applied where the electron affinity lies below the target work function. This is designed to favour higher electron affinities, to match the semiconductor to less reactive, higher work function metals. In this work, we use *W* = 4.1 eV to match metals such as Ag, Cu and Au, with work functions of 4.26, 4.65 and 5.1 eV respectively.

Both fitness functions were evaluated for each molecule generated by the evolutionary algorithm using calculations at the B3LYP/6-311+G** level of theory using GAUSSIAN09.^21^ Solid-state electron affinities were approximated from calculated gas phase adiabatic electron affinities by taking advantage of the known linear correlation between the two quantities.^22,23^ The relationship was calibrated for 12 molecules against experimental low-energy inverse photoemission spectroscopy (LEIPS) values for thin-films organic semiconductors; see ESI† for details.

### 2.4 Crystal structure prediction

CSP was carried out for the most promising molecules identified from the evolutionary search, using the Global Lattice Energy Explorer (GLEE) program.^24^ The searches used a low-discrepancy, quasi-random sampling of crystal packing variables to uniformly sample the lattice energy surface of each molecule in the most frequently observed space groups for organic molecules. Local energy minimisation was applied to all trial crystal structures using an empirically parameterised atom–atom force field with electrostatic interactions described by an atomic multipole electrostatic model based on the calculated molecular charge densities.

### 2.5 Electron mobility calculations

Electron mobility calculations were performed on all predicted crystal structures that are within 7 kJ mol^−1^ of that molecule's global lattice energy minimum. This energy window is chosen to include most experimentally observable structures, based on the distribution of calculated energy differences between known polymorphs.^25^ Mobility calculations used a hopping transport model with charge carriers localised to a single molecule centred at the molecular centroid. Hopping rates were calculated using Marcus theory,^26^4
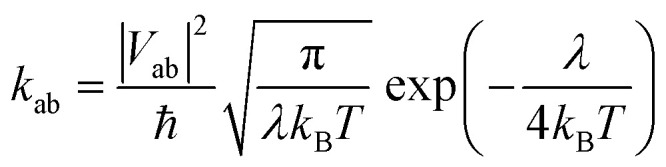
where electron reorganisation energies were evaluated during the evolutionary optimisation. Electronic couplings, |*V*_ab_|, between molecules were determined using the FODFT approach with PBE/TZ2P, as implemented in the ADF program.^27,28^ All calculated electronic couplings were scaled by 1.325 to bring FODFT values in-line with high-level *ab initio* calculations.^29,30^

A hopping transport network was generated by first designating each molecule in the unit cell with a site label. Hopping rates were calculated for all dimers with at least one atom–atom distance shorter than the sum of each van der Waals radii plus 1.5 Å from each site. The total number of dimer evaluations required was reduced within a crystal structure by finding identical dimers using the Kabsch algorithm^31^ with a RMSD threshold of 0.001 Å and only evaluating them once. The hopping rates are determined for a given site to the same site in an adjacent unit cell or a different site in the same or adjacent unit cell. A hopping transport network therefore includes details of the hopping rate, displacement vector and its start and end sites.

Using the generated hopping transport network, kinetic Monte Carlo simulations with the rejection-free procedure^32^ were carried out using in-house developed code to determine the diffusion tensor. Diffusion tensor elements were averaged over 100 000 trajectories with 1000 iterations per trajectory. The mobility tensor elements were then obtained with the Einstein relation *μ*_αβ_ = *qD*_αβ_/*k*_B_*T*. A temperature of 300 K was used in all rate and mobility calculations.

Marcus theory is not expected to provide a quantitative assessment of carrier mobilities for small molecule semiconductor materials.^12,33–35^ The intention here is to use charge mobilities obtained using Marcus theory as an inexpensive descriptor to favour crystal structures with low reorganisation energies, large electronic couplings and sufficiently connected pathways for charge transport through the crystal structure. Using Marcus theory in this manner is similar to other recent high throughput methods which have evaluated structures using these types of properties.^11^ As an assessment of its predictive power against a more complete description of charge transport, we carried out comparisons of Marcus theory against mobilities from non-adiabatic molecular dynamics^36–41^ (see Table S2 and Fig. S4, ESI† for details) for a series of functionalised tetracenes.^41^ These results indicate a good correlation for the majority of structures across the range of mobilities, but occasional outliers where Marcus theory predictions are poor. Our intention here is to present the framework of the evolutionary material discovery approach within which the simple charge transport model can be replaced when new methods become available at an affordable computational cost.

## 3 Results and discussion

### 3.1 Minimisation of the electron reorganisation energy

Ten runs of the evolutionary algorithm were performed with the target of minimising the electron reorganisation energy (fitness function *F*_A_). We expected the global minimum of *F*_A_ within the chemical space considered to correspond to pentacene—any aza-substitution or non-linearity of arrangement of rings was expected to disrupt delocalisation of the excess electron, leading to an increase in the reorganisation energy. This was confirmed by the results, in which no molecules could be located with lower *F*_A_ than pentacene after extensive searches. The known global minimum aided the analysis of performance of the search, which was used in developing the algorithm: testing of population sizes, types of transformations and their probabilities.

**Fig. 4:**
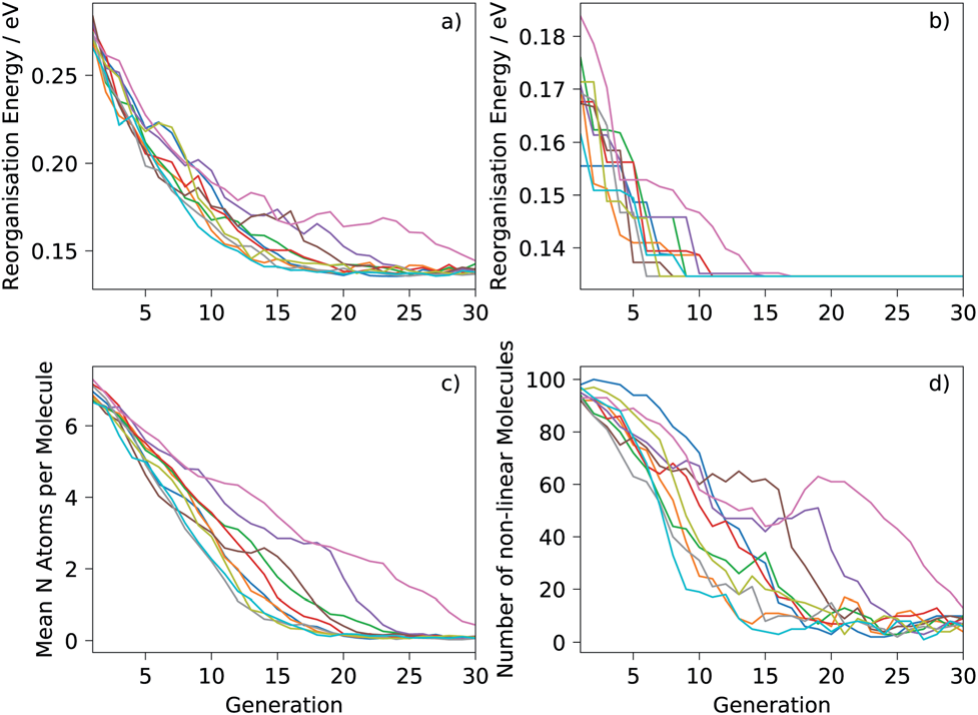
Progress of the ten runs of the evolutionary algorithm, each displayed as a different colour: (a) mean reorganisation energy of the population; (b) minimum reorganisation energy; (c) mean number of nitrogen atoms per molecule; (d) number of non-linear molecules in the population. The population size was 100 in all runs.

The mean reorganisation energy of the population of molecules decreased steadily during the initial generations and at a similar rate in each of the ten runs (Fig. 4a). Nine of the ten runs converge to a similar mean by 20–25 generations. Progress towards the global minimum was quicker: the minimum reorganisation energy within the population was observed to decrease rapidly (Fig. 4b), finding the same global minimum—pentacene—in each run. The location of pentacene required between 6 to 17 generations (Table 1). This variation between runs is expected due to the inherent randomness in the search algorithm and of the initial population of molecules. However, the number of molecules sampled until the global minimum was located showed less variation (Table 1) and, in the worst case, involved calculations on 1.6% of molecules in the chemical space considered here. This demonstrates large efficiency gains for the evolutionary search over a random search of molecules from the chemical space.

**Table 1:** Numbers of evolutionary generations and unique molecules sampled before locating pentacene, the global minimum in electron reorganisation energy. The total chemical space includes 68 064 molecules

Run	Number of generations	Molecules sampled	Proportion of chemical space sampled
1	9	642	0.94%
2	11	745	1.09%
3	9	672	0.99%
4	11	778	1.14%
5	15	1035	1.52%
6	8	572	0.84%
7	17	1110	1.63%
8	6	420	0.62%
9	7	513	0.75%
10	9	631	0.93%

**Fig. 5:**
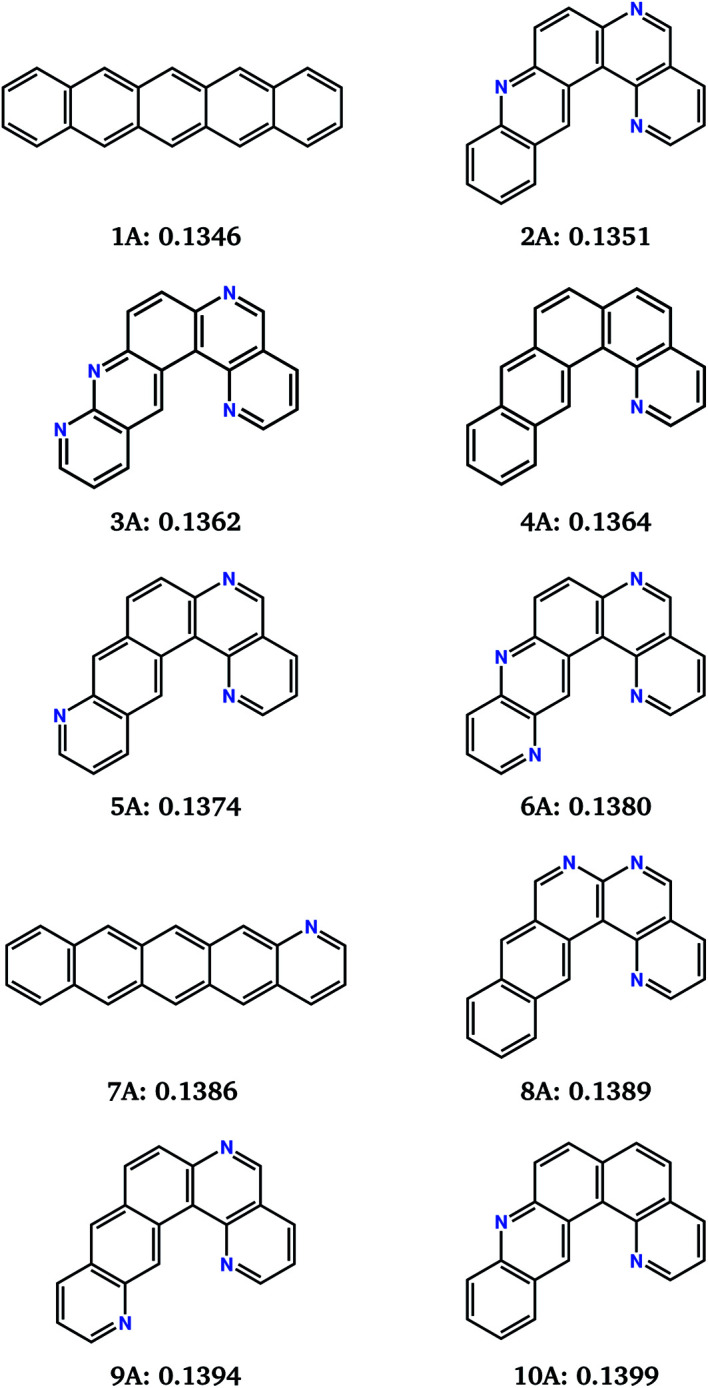
Chemical diagrams of the top 10 best performing molecules from the combined 10 runs of the evolutionary search for the minimisation of the electron reorganisation energy (fitness function *F*_A_). Name labels and *F*_A_ fitness values (in eV) are given below each chemical diagram.

Fig. 4c and d show how the chemistry of the population of molecules evolves during the search. The randomisation process produces an initial population with a large number of nitrogen atoms per molecule and over 90% of molecules in the initial population are non-linear. As expected, the fitness function that only considers the reorganisation energy favours less nitrogen substitution: the populations converge to almost completely unsubstituted PAHs (Fig. 4c). Non-linearity of the fused ring system (as defined in the ESI†) is also generally disfavoured and decreases through each run, but with greater variability between runs and some periods where the number of non-linear molecules increases for several generations (Fig. 4d). This behaviour is indicative of having found favourable non-linear configurations. Some runs keep a large proportion of non-linear molecules in the population until well past the point where the minimum has been located. In fact, we find that most of the molecules just above pentacene in reorganisation energy contain the same angularly fused core ring structure – see Fig. 5.

This naphtho[1,2-*a*]anthracene motif was unanticipated, but dominates the low reorganisation energy region of chemical space. 8 of the best 10 molecules located by the 10 combined searches contain the same core structure. These molecules differ in their level and pattern of aza-substitution, but all have the same nitrogen in the inner curved, bay region^20^ of the molecule. The resulting N…H–C interaction stabilises the planar molecular structure, which is presumably favourable for delocalisation of the excess electron.

The identification of this structural motif with reorganisation energies almost as low as pentacene demonstrates the usefulness of the evolutionary search for suggesting previously unexplored molecules as promising synthetic targets. The low sensitivity of *λ*_−_ to the placement of additional nitrogens (molecules **2A–6A** and **8A** to **10A**, Fig. 5) suggests that molecules with this core can be functionalised to control their crystal packing without sacrificing their inherent low reorganisation energy.

Reorganisation energies of the top 10 molecules identified over all 10 evolutionary searches (labelled **1A–10A**, Fig. 5) show a negligibly small variation, ranging from 0.1346 to 0.1399 eV. Therefore, differences in electron mobilities within the crystal structures of the best molecules located by the search will be entirely determined by the electron coupling between molecules, due to their crystal packing. Charge carrier transport in pentacene is known to be limited by its herringbone crystal packing,^7,42^ with molecules arranged edge-to-face. Aza-substitution has been shown to modify the preferred packing^7^ by introducing weak hydrogen bonding. Combined with their shape difference, this should lead to different crystal packing preferences within the other top-10 molecules.

### 3.2 Property maps of chemical space

**Fig. 6:**
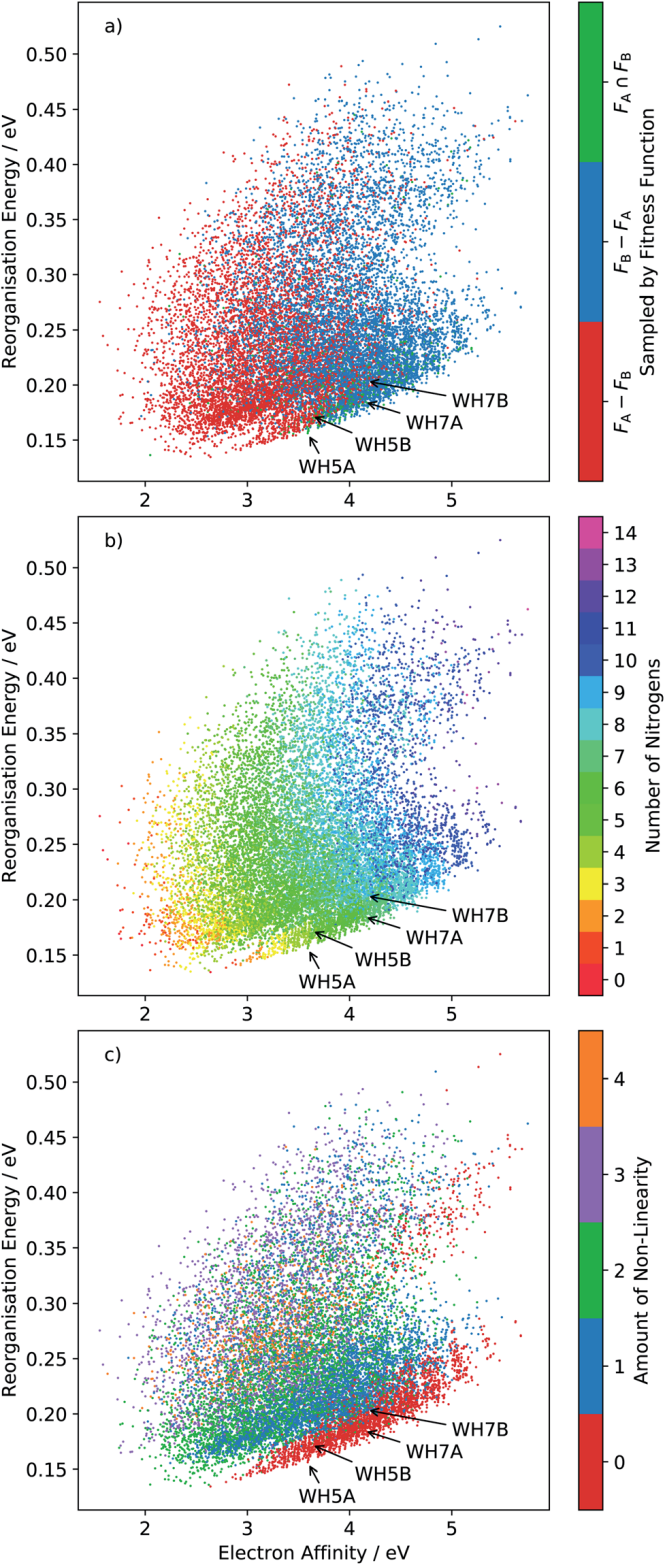
Plots of the reorganisation energy and solid-state electron affinity for all molecules sampled across all 20 runs of the evolutionary algorithm, minimising fitness function *F*_A_ (10 runs) and *F*_B_ (10 runs). A total of 15 870 unique molecules (23.3% of the total chemical space) are sampled in this combined set of searches. Points are plotted with three different colour series: (a) molecule sampled by fitness function *F*_A_ and not *F*_B_ (*F*_A_ − *F*_B_), *F*_B_ and not *F*_A_ (*F*_B_ − *F*_A_) or *F*_A_ and *F*_B_ (*F*_A_ ∩ *F*_B_); (b) colour coded by number of nitrogen atoms in the molecule and (c) by degree of non-linearity in the molecular structure (as defined in the ESI†). Locations of four azapentacenes molecules proposed by Winkler and Houk^15^ (**WH5A**–**WH7B**) are labelled on each plot.

The distribution of all sampled molecules (including evolutionary searches minimising fitness function *F*_A_ and those minimising *F*_B_, discussed below) is shown in Fig. 6. This reorganisation energy-electron affinity map of the chemical space highlights a competing trend between minimisation of the reorganisation energy for electron transport and the need for a high electron affinity for an n-type semiconductor. The best molecules are expected to lie along the low-*λ*_−_ edge of the distribution – the Pareto set of this multi-objective optimisation – along which an increase in electron affinity comes at a cost of increasing the reorganisation energy.

For this reason, the searches using fitness function *F*_A_ preferentially sampled the low-electron affinity regions of chemical space (Fig. 6a). The best molecules identified according to *F*_A_ all have electron affinities in the range 2.0–2.8 eV, near the calculated electron affinity of pentacene (2.64 eV), which is close to the experimental values of 2.35, 2.70 and 3.14 eV for three different molecular orientations of pentacene crystalline films.^43–45^ Although pentacene-based OFETs more commonly result in p-type behaviour, the behaviour can be controlled by selecting electrodes with a work function that matches the semiconductor's ionisation energy or electron affinity. In fact, pentacene has reported ambipolar or n-type behaviour on low work function metals.^46,47^ Therefore, to reduce the barrier for electron injection in the *F*_A_ set of molecules and achieve an n-type OFET, a low work function electrode such as calcium (*W* = 2.87 eV) would be required. The discovery of molecules with simultaneously low reorganisation energy and high electron affinity, to match more typical metal electrodes, requires a multi-objective optimisation, which we address through linear scalarisation, leading to fitness function *F*_B_, whose results are discussed below.

These property maps of chemical space also reveal important chemical trends that can inform molecular design. From Fig. 6b we can see that there is a general increase in reorganisation energy and electron affinity with the number of nitrogen substitutions. We also observe a discontinuity in the lower edge of the distribution, where there is a clear shift in a large group of molecules towards higher electron affinities. Comparison with Fig. 6c shows that this region corresponds to linear molecules, in which no bends have been introduced into the ring arrangement of the pentacene core. Linear molecules dominate the low reorganisation energy region of chemical space for electron affinities larger than around 2.6 eV. The trend amongst non-linear molecules is less clear than with nitrogen substitution, such that the property distributions of molecules with 1, 2, 3 and 4 degrees of non-linearity overlap significantly.

**Fig. 7:**
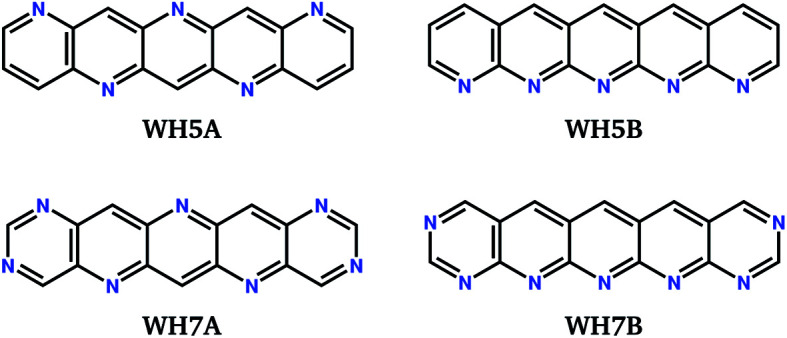
Chemical diagrams of four azapentacenes proposed by Winkler and Houk.^15^

We label in Fig. 6 the positions of four molecules proposed by Winkler and Houk^15^ (**WH5A**–**WH7B**, Fig. 7) based on calculations of their single molecule electronic properties, with the aim of minimizing the reorganization energies whilst targeting gas phase electron affinities above 3 eV. All four molecules are linear azapentacenes so lie within the linear-molecule region of Fig. 6c, and could lead to good electron mobilities due to the relatively small differences between reorganisation energies within this region. We see that **WH5A** was particularly well designed and lies on low-*λ*_−_ edge of the distribution. We take these molecules, proposed by experienced chemists using computational tools and intuition about crystal packing, as a benchmark for the best molecules proposed by our evolutionary algorithm.

### 3.3 High electron affinity aza-substituted candidates

Ten runs of the evolutionary algorithm were performed to minimise fitness function *F*_B_, with all other details of the search identical to those using *F*_A_. *F*_B_ includes a linear penalty equal to the Schottky–Mott model of the barrier for electron injection, which is applied to molecules with solid state electron affinities below 4.1 eV. The impact on the search is to restrict most sampling to molecules in the high electron affinity region of chemical space (Fig. 6a). In this region, all low-reorganisation energy molecules are linear (Fig. 6c) with 6 or 7 nitrogens (Fig. 6b), differing in the pattern of nitrogen substitution.

**Fig. 8:**
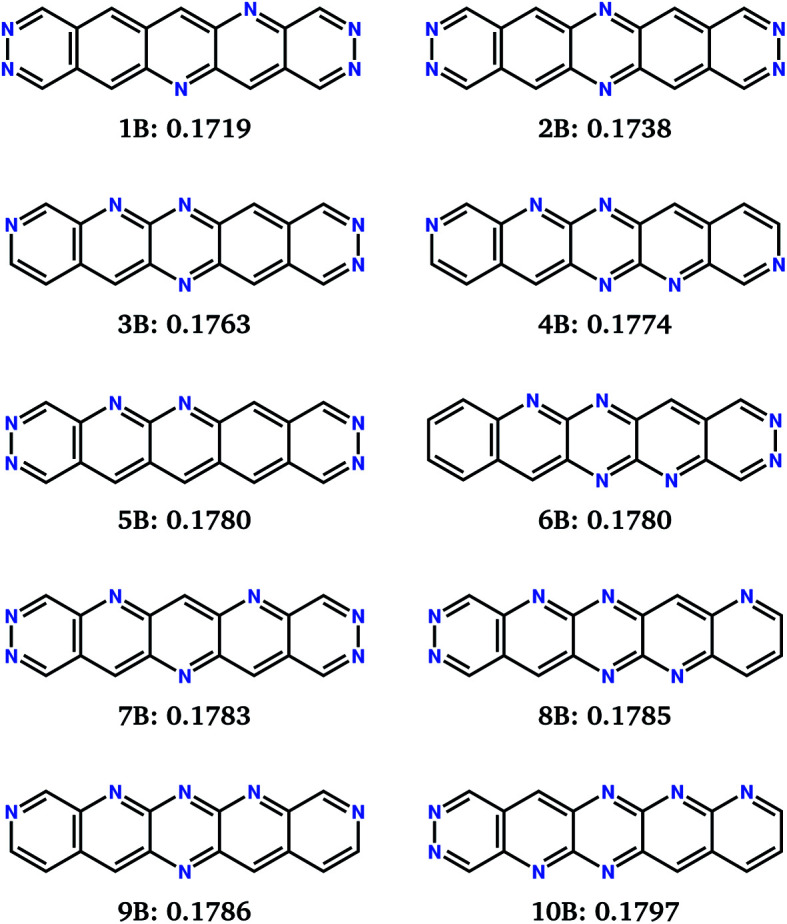
Chemical diagrams of the top 10 best performing molecules from the combined 10 runs for the evolutionary search for minimisation of fitness function *F*_B_. Labels and *F*_B_ fitness values (in eV) are given below each chemical diagram.

The 10 best molecules from these searches are shown in Fig. 8; we label these **1B** to **10B**. Double nitrogen substitution of the terminal rings, leading to pyridazine functionality, emerges from these searches as being particularly favoured. Pyridazine rings have gained some interest in π-conjugated materials^48^ and polymer thin-film field effect transistors.^49^ Our results suggest that this is a globally optimum solution for combining low electron reorganisation energy with high electron affinity in aza-substituted acenes. Pyridazine groups occur at both ends of the two best molecules according to *F*_B_ (**1B** and **2B**), as well as **5B** and **7B**; only two of the top 10 (**4B** and **9B**) lack a pyridazine ring. The remaining nitrogens decorate the long edges of the molecules in a variety of symmetric and asymmetric patterns. Our previous work has shown that nitrogens along the long edge of pentacene can favour sheet-like crystal packing,^7^ often leading to improved electron mobility.

The *F*_B_ set of molecules have estimated electron affinities from 4.1 to 4.3 eV, matching the work function targeted by the fitness function and similar to the electron affinities of commonly used n-type materials, such as C_60_ and C_70_ (ref. 50) (∼4 eV).^51^ Thus, they are more suitable as electron transport materials for n-channel OFETs using more typically used electrodes, *e.g.* gold (*W* = 5.1 eV).

### 3.4 Energy structure function maps

To estimate the electron mobility achievable by each molecule, we need both the molecular electronic properties, which were optimised during the evolutionary search, as well as its likely crystal structures. We therefore preformed CSP for the top ten best performing molecules obtained from each of fitness functions *F*_A_ and *F*_B_ and the four benchmark molecules, **WH5A**–**WH7B**. The mean electron mobility for each crystal structure within 7 kJ mol^−1^ from the global minimum on each molecule's landscape was obtained from the trace of the calculated mobility matrix, *μ̄* = tr(*μ*)/3.

We have previously discussed several measures for assessing a molecule based on the properties calculated for its ensemble of low energy crystal structures.^7,8^ A central assumption of CSP is that the most likely observable crystal structure is the structure with the lowest calculated energy. If the energy model used in CSP is reliable, then the calculated mobility for this global lattice energy minimum structure, *μ̄*_GM,_ is a simple first measure of each molecule's expected performance.

**Fig. 9:**
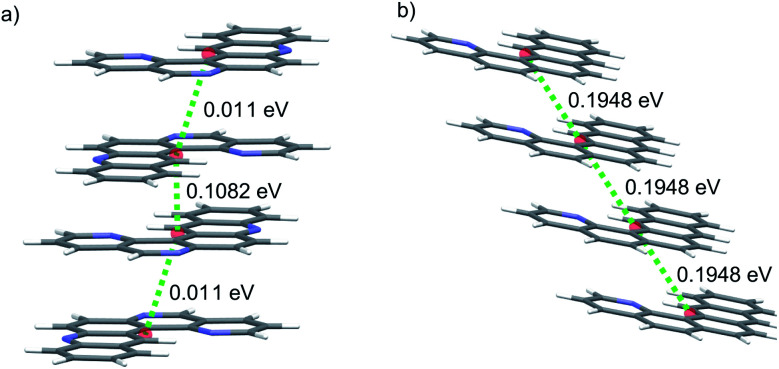
Coplanar stacking of molecules in the global energy minimum predicted crystal structures of molecules (a) **2A** and (b) **4A**. Red spheres mark the centroids of each molecule. Green dotted lines are the hopping pathways along molecular stacks, showing values of the electronic coupling, |*V*_ab_|, after scaling.

We first consider molecules **1A–10A** (Fig. 5), optimised with respect to reorganisation energy: their electron mobilities show a large variability (Table 2), ranging from less than 1 cm^2^ (Vs)^−1^ for molecule **2A** up to 17 cm^2^ (Vs)^−1^ for **4A** – the singly nitrogen substituted naphtho[1,2-*a*]anthracene. The differences in *μ̄*_GM_ amongst such similar molecular structures show the large effect of crystal packing preference on the charge carrier mobility, despite similarly small reorganisation energies. The global minimum crystal structures of both **2A** and **4A** feature co-planar molecular stacking in the so-called γ packing of PAH molecules,^52^ which is usually considered to promote high mobility. However, while the stacked molecules are orientationally aligned in the predicted structure of **4A** (Fig. 9b), the molecules alternate orientation along the molecular stacks for **2A** (Fig. 9a), which likely disrupts electronic coupling and leads to its poor electron mobility. Considering only the properties of their global energy minimum crystal structure, molecules **3A**, **4A** and **8A** are the most attractive targets for synthesis and characterisation. All three have predicted lowest energy crystal structures featuring stacks of orientationally aligned molecules, as in Fig. 9b.

**Table 2:** Summary of the electron transport properties for the top 10 molecules identified through evolutionary optimisation of fitness functions *F*_A_ (**1A–10A**), *F*_B_ (**1B–10B**) and four azapentacenes proposed by Winkler and Houk^15^ (**WH5A**–**WH7B**): the number of structures with a lattice energy within 7 kJ mol^−1^ of the global minimum in the CSP landscape; the global minimum, average and deviation of the mean electron mobility; the reorganisation energy for electron transport and solid-state electron affinities. Solid-state electron affinities were estimated from the gas phase isolated molecule calculations – see ESI†.

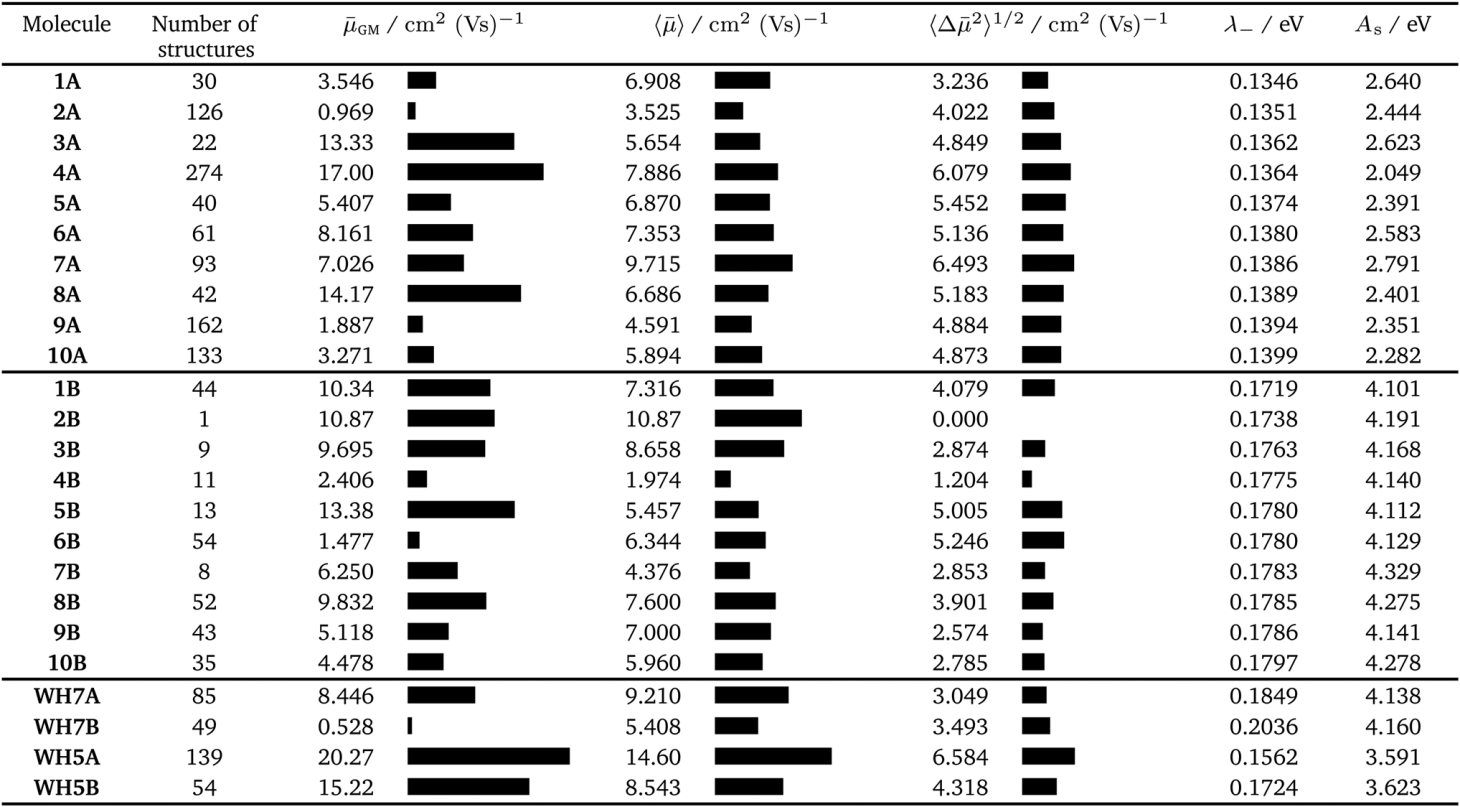

The number of predicted crystal structures in the low energy region of the landscape varies greatly between molecules (Table 2), and corresponds to small energy differences between predicted structures in almost all cases. To better reflect uncertainties in the energetic ranking of structures, due to errors in the energy model and the lack of temperature in our crystal structure evaluation,^25,53^ as well as uncertainties related to kinetic influences on crystallisation outcome, we have previously proposed a probabilistic view of each molecule's ESF map. For this, we calculate a Boltzmann-like weighted landscape average of the electron mobility for the predicted crystal structures:5
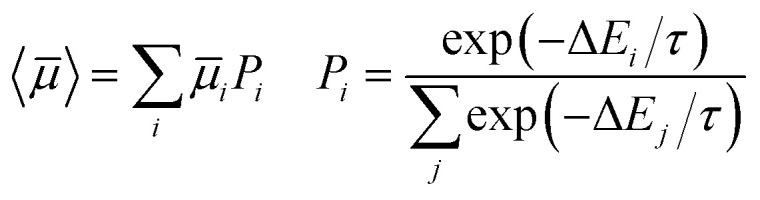
where Δ*E*_*i*_ is the energy difference of a crystal structure from the global minimum; this is used to assign a probability, *P*_*i*_, that this crystal structure will be observed experimentally. The constant *τ* = 2.70 kJ mol^−1^ was obtained by fitting to energy differences between known pairs of polymorphs.^7,25^

**Fig. 10:**
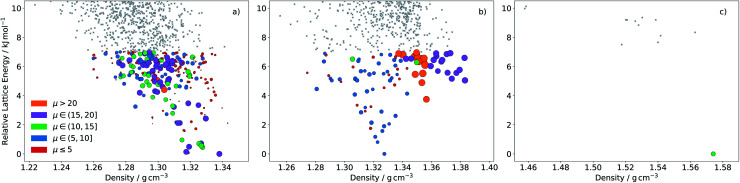
ESF map of electron mobility in the predicted crystal structures of molecules (a) **4A**, (b) **7A** and (c) **2B**. Each point corresponds to a distinct predicted crystal structure. Colouring and size of the circles correspond to the magnitudes of the calculated mean electron mobilities (in cm^2^ (Vs)^−1^). Grey points are structures above 7 kJ mol^−1^, for which mobilities were not calculated.

Naturally, molecules are less distinguished using the landscape averaged mobility than that based on one crystal structure. Molecule **4A** is still ranked highly based on 〈*μ̄*〉, due to a large number of high mobility structures on its ESF map (Fig. 10a), while **7A** now also ranks highly. The high 〈*μ̄*〉 for **7A** results from a large family of high density crystal structures with very high mobility between 4 and 7 kJ mol^−1^ above the global minimum (Fig. 10b). Although the average mobility is high over the low energy predicted crystal structures, such a target represents a risk: the landscape contains large numbers of both high and low mobility crystal structures.

A wide range of properties within the energetic region of possible crystal structures corresponds to a large uncertainty in the target property. To capture the risk associated with uncertainty in the resulting crystal structure, we propose a measure of the variability of the mobility amongst the predicted structures:6
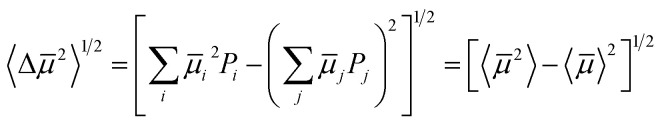
where *P*_*i*_ is calculated in the same way as in the landscape-averaged mobility. 〈Δ*μ̄*^2^〉^1/2^ approaches zero for a landscape of crystal structures with uniform mobility.

An ideal target molecule should maximise 〈*μ̄*〉, while minimising 〈Δ*μ̄*^2^〉^1/2^. However, for molecules **1A–10A**, we find that the two measures have similar magnitudes; some of these molecules could lead to materials with very high electron mobility, but the risk is high that synthesis could lead to a low mobility material.

Molecules **1B–10B** have electron affinities that are better suited for n-type behaviour and, despite their higher reorganisation energies, yield crystal structures with predicted mobilities in the same range as **1A–10A** (Table 2). Several of the pyridazine-based pentacene derivatives (*e.g.***1B**, **2B** and **3B**) show promising predicted properties, based on their global minimum crystal structures (*μ̄*_GM_) and landscape-averaged mobilities (〈*μ̄*〉). These higher electron affinity molecules also show less variability in electron mobility, 〈Δ*μ̄*^2^〉^1/2^, than **1A–10A**. In particular, **2B** leads to a sparse crystal structure landscape (Fig. 10c) with an unusually large (∼8 kJ mol^−1^) energy gap between the global minimum and all higher energy predicted crystal structures; this gives a high confidence of observing the low energy predicted crystal structure, so that 〈Δ*μ̄*^2^〉^1/2^ = 0 and 〈*μ̄*〉 = *μ̄*_GM_ is the highest landscape-averaged electron mobility of all the molecules.

**Fig. 11:**
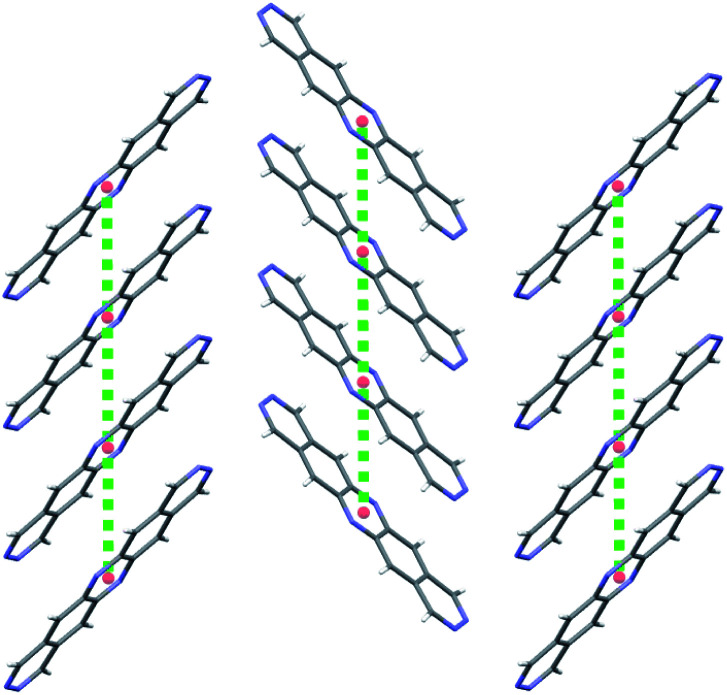
A 2D plane of the global minimum energy crystal structure of **2B** showing γ-type stacking of molecules. Red spheres mark the centroids of each molecule and the green dotted lines are the hopping pathways with the largest rate of electron transport in this crystal structure.

**2B** is therefore the most promising of the molecules identified in this study, and an attractive option for synthesis and characterisation as well as further, more detailed computational studies, such as extended CSP in further space groups and higher level assessment of charge carrier mobility. At the current level of theory applied to the structure and property calculations, the global lattice energy minimum structure of **2B** has a mobility tensor with eigenvalues of 30.18, 2.12 and 0.30 cm^2^ (Vs)^−1^, therefore exhibiting predominantly 1D conduction. Inspection of the crystal structure and electronic coupling of its dimers shows that conduction occurs along the molecular stacks, along which there is large electron coupling (|*V*_ab_| = 0.1911 eV, after scaling) between molecules, larger than any other direction by an order of magnitude for this crystal; the 1D electron hopping pathway in this crystal structure is shown in Fig. 11.

Finally, we ask how the molecules arrived at by the evolutionary approach compare to those designed through intuition by experienced chemists. The predicted properties for molecules **WH5A**–**WH7B** are included in Table 2. **WH7A** and **WH7B** are most directly comparable to those optimised to fitness function *F*_B_, as their electron affinities fall within the range covered by **1B–10B**. **WH7A** compares well to the optimised molecules, but is inferior to molecule **2B** in all of its properties. **WH7B** is out-performed by most of the molecules proposed by the evolutionary algorithm. Molecules **WH5A** and **WH5B** are less directly comparable, as their electron affinities lie between those in sets **1A–10A** and **1B–10B**. However, they have very good predicted electron mobilities, particularly of their global minimum energy predicted crystal structures. **WH5A** has a higher *μ̄*_GM_ and 〈*μ̄*〉 than any of the molecules proposed by the evolutionary optimisation, albeit with higher variability 〈Δ*μ̄*^2^〉^1/2^, and hence risk, than all of the molecules proposed by the evolutionary approach. The good properties of **WH5A** and **WH7A** are due, in part, to the crystal packing; the intermolecular hydrogen bonding, which leads to stacking of molecules in many of the low energy predicted crystal structures, was correctly anticipated by Winkler and Houk.^7,15^

The comparison between **WH5A**–**WH7B** and the molecules proposed by the evolutionary algorithm underscores one of the main weaknesses of our current approach: while chemists can develop useful intuition about crystal packing, our evolutionary optimisation is currently ‘blind’ to the likely crystal structures of each molecule, because CSP is performed after evolutionary optimisation. This points to a challenging future development of the method: to include CSP within the fitness function evaluation itself. Evaluation of predicted crystal structures within the evolutionary search might also differentiate molecules more clearly, whereas the current evaluation yields large numbers of molecules with small differences in reorganisation energy that can be overridden by differences in crystal packing.

The comparison also highlights a strength of the evolutionary algorithm: multi-objective optimisation, *e.g.* for low reorganisation energy and high electron affinity, is challenging for intuitive molecular design, particularly for more complex molecules, where the influence of molecular structure on crystal packing will be less clear. However, multi-objective optimisation can be performed in an algorithmic search, such as with the simple approach that we took here with fitness function *F*_B_.

## 4 Conclusions

We have demonstrated how coupling of an evolutionary optimisation algorithm for chemical space exploration with crystal structure prediction and property simulations can be a powerful approach for discovery of functional molecular crystals. The method is designed to assist in the discovery of molecular materials where the structure–property relationships are not obvious and intuitive.

Here, the methodology has been applied to the relatively small chemical space of aza-substituted pentacenes, for the identification of promising n-type semiconductor materials. The evolutionary algorithm is flexible in the choice of fitness function used to guide optimisation. Two simple measures of molecular fitness are used here, both chosen to maximise the probability for large electron mobilities. The first minimises the electron reorganisation energies from Marcus theory and a second fitness that combines low reorganisation energy with high electron affinity, to decrease the barrier for the injection of an electron into the semiconductor and increase the overall OFET performance.

The evolutionary search, which is driven by a set of molecular transformation operations, is found to efficiently identify the fittest molecules – here, typically requiring calculations on 1 percent of molecules in the chemical space considered. The searches have identified promising chemical substructures: apart from pentacene, the region of lowest reorganisation energy is dominated by molecules featuring the naphtho[1,2-*a*]anthracene motif, whose electronic properties (electron reorganisation energy and electron affinity) show low sensitivity to further functionalisation – here, further nitrogen substitution. Several of these molecules yield global energy minimum crystal structures with very high predicted electron mobilities. For high electron affinity, as well as low reorganisation energy, we find that a linear pentacene core with terminal pyridazine rings is common amongst many of the best molecules.

While optimisation of molecular properties is easily implemented and computationally efficient, we find that the influence of crystal packing has a dominant role in determining electron mobility through its impact on electronic coupling between molecules; there is a large variation in calculated mobility of crystals predicted for molecules of nearly equal reorganisation energies, as well as between low energy predicted crystal structures of the same molecule. For this reason, future development of the evolutionary optimisation for molecular materials should include crystal packing effects within the fitness function. This is challenging because of the computational cost associated with CSP, but developments such as machine learned energy models^54,55^ and fast structure searching algorithms could help reduce these timescales.

CSP introduces a complication to the evaluation of molecules because each molecule is associated with an ensemble of crystal structures of similar energetic stability, but sometimes large variation in properties. We use several measures to judge the fitness of a molecule's landscape of predicted crystal structures, based on properties of the lowest energy structure, a weighted average over low energy structures, and assessment of the variability of properties between crystal structures. These provide measures of the potential of a molecule, as well as risk associated with uncertainty of the resulting crystal structure.

Molecules with large landscape-averaged properties as well as small variation in properties between low energy potential crystal structures are attractive. Small variation in properties can result from a sparsity in the crystal structure landscape, a further advantage of which is that small numbers of structures can be treated with more rigorous methods for property prediction. From this work, the symmetric hexa-azapentacene molecule, **2B**, meets these criteria, with a large energy gap between predicted crystal structures and a high calculated electron mobility for the lowest energy structure.

Comparison was made to a series of azapentacenes previously proposed as promising n-type organic semiconductors, **WH5A**, **WH5B**, **WH7A** and **WH7B**, none of which were found to have both a large average and small variation of the electron mobilities, properties which we predict for molecule **2B**. From this comparison, we judge that the evolutionary algorithm developed here is at least as successful as intuitive molecular design assisted with computational tools, while also having clear opportunities for development, particularly through integration of solid state structure prediction more strongly into the evolutionary process itself.

## Conflicts of interest

There are no conflicts to declare.

## Supplementary Material

SC-011-D0SC00554A-s001

SC-011-D0SC00554A-s002

SC-011-D0SC00554A-s003

SC-011-D0SC00554A-s004

SC-011-D0SC00554A-s005

SC-011-D0SC00554A-s006

SC-011-D0SC00554A-s007

SC-011-D0SC00554A-s008

SC-011-D0SC00554A-s009

SC-011-D0SC00554A-s010

SC-011-D0SC00554A-s011

SC-011-D0SC00554A-s012

SC-011-D0SC00554A-s013

SC-011-D0SC00554A-s014

SC-011-D0SC00554A-s015

SC-011-D0SC00554A-s016

SC-011-D0SC00554A-s017

SC-011-D0SC00554A-s018

SC-011-D0SC00554A-s019

SC-011-D0SC00554A-s020

SC-011-D0SC00554A-s021

SC-011-D0SC00554A-s022

SC-011-D0SC00554A-s023

SC-011-D0SC00554A-s024

SC-011-D0SC00554A-s025

SC-011-D0SC00554A-s026

SC-011-D0SC00554A-s027

SC-011-D0SC00554A-s028

SC-011-D0SC00554A-s029

SC-011-D0SC00554A-s030

SC-011-D0SC00554A-s031

SC-011-D0SC00554A-s032

SC-011-D0SC00554A-s033

SC-011-D0SC00554A-s034

SC-011-D0SC00554A-s035

SC-011-D0SC00554A-s036

SC-011-D0SC00554A-s037

SC-011-D0SC00554A-s038

SC-011-D0SC00554A-s039

SC-011-D0SC00554A-s040

SC-011-D0SC00554A-s041

SC-011-D0SC00554A-s042

SC-011-D0SC00554A-s043

SC-011-D0SC00554A-s044

SC-011-D0SC00554A-s045

SC-011-D0SC00554A-s046

SC-011-D0SC00554A-s047

SC-011-D0SC00554A-s048

SC-011-D0SC00554A-s049

SC-011-D0SC00554A-s050

SC-011-D0SC00554A-s051

SC-011-D0SC00554A-s052

SC-011-D0SC00554A-s053

SC-011-D0SC00554A-s054

SC-011-D0SC00554A-s055

SC-011-D0SC00554A-s056

SC-011-D0SC00554A-s057

SC-011-D0SC00554A-s058

SC-011-D0SC00554A-s059

SC-011-D0SC00554A-s060

SC-011-D0SC00554A-s061

SC-011-D0SC00554A-s062

SC-011-D0SC00554A-s063

SC-011-D0SC00554A-s064

SC-011-D0SC00554A-s065

SC-011-D0SC00554A-s066

SC-011-D0SC00554A-s067

SC-011-D0SC00554A-s068

SC-011-D0SC00554A-s069

SC-011-D0SC00554A-s070

SC-011-D0SC00554A-s071

SC-011-D0SC00554A-s072

SC-011-D0SC00554A-s073
