# Supplementary material for: Evolutionary chemical space exploration for functional materials: computational organic semiconductor discovery
Source: Chem Sci. 2020 Apr 22;11(19):4922–33. doi: 10.1039/d0sc00554a (PMC8159259; doi:10.1039/d0sc00554a)
Supplement: SC-011-D0SC00554A-s073 [file SC-011-D0SC00554A-s073.pdf]

Electronic Supplementary Material (ESI) for Chemical Science.
This journal is © The Royal Society of Chemistry 2020

Supplementary Information for: Evolutionary Chemical Space Exploration for Functional Materials: Computational Organic Semiconductor Discovery

Chi Y. Cheng, Josh E. Campbell and Graeme M. Day

S1 Full details of the evolutionary search algorithm

A flexible evolutionary search algorithm was developed for the global optimisation of a molecule's chemical structure for a given calculated fitness function. The region of chemical space that will be searched and the possible moves that can be made across chemical space by the algorithm are defined by three input variables and four transformation operations.

The three input variables—**`smiles`**, **`smarts`** and **`molsize`**—define molecular fragments that can be used by the algorithm to build or modify molecules. **`smiles`** contains a list of SMILES strings[1, 2] representing molecules or fragments, acting as the primary building blocks for the creation of larger molecules. Strings containing asterisks represent fragments while those without represent base molecules. The asterisks in the SMILES string such as *`'c1c**cc1'`* denote the attachable positions of that fragment onto another molecule. **`smarts`** is a list of SMARTS strings[3] which are used for fragment matching and mutations. **`molsize`** contains the limits on the size of molecules that can be created where, for this work, we define molecular size by the number of rings contained in that molecule.

The four transformation operations—**`Addition`**, **`Crossover`**, **`Recombination`** and **`Mutation`**—act by modifying one or more molecules (Fig. S1). **`Addition`** transforms a molecule into a larger molecule by the attachment of a fragment to a random position on that molecule. The transformation operates by first randomly selecting a possible bonding position on the molecule and a orientation of the fragment. The molecule and fragment are then added together to create a larger molecule (Fig. S1a). **`Crossover`** fragments two parent molecules at a random position into two parts giving a total of four fragments. Two child molecules are then generated by combining two fragments (one from each parent) together (Fig. S1b). **`Recombination`** fragments a single molecule at a random position. The fragments are combined back together after moving the fragmented positions, generating an isomer of the initial molecule (Fig. S1c). In **`Mutation`**, a position on the molecule that is matched by any SMARTS string from the **`smarts`** list variable is randomly selected and replaced by a different fragment randomly selected from the same list (Fig. S1d).

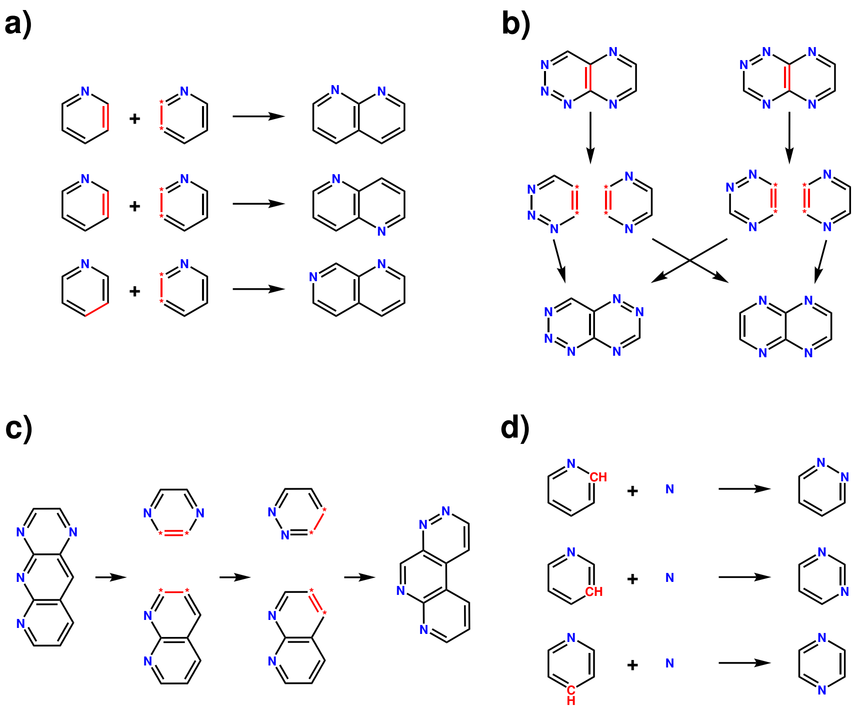

Figure S1: Examples of the four transformation operations implemented in the evolutionary algorithm. a) The **`Addition`** operation, illustrated with three possibilities for the addition of a pyridine fragment to a pyridine molecule, forming a naphthyridine type molecule; b) **`Crossover`** between two aza-napthalene molecules showing one crossover possibility for the example parent molecules. Additional possibilities can occur due to freedoms in the orientation of the fragments when combined together and the possible pairings of each fragment. c) The **`Recombination`** transformation of an aza-anthracene molecule, creating an isomer of the initial molecule. Additional possibilities can occur due to freedoms in the fragmentation positions, fragmentation position moves and the orientation of the fragments when combined together. d) The **`Mutation`** transformation on the pyridine molecule with a nitrogen atom fragment, showing three possible mutations forming either a pyridazine, pyrimidine or pyrazine molecule.

When starting the evolutionary search algorithm an initial population of randomly generated molecules are created using the input variables and transformation operations. Randomised molecules are created by randomly selecting one of the base molecules from the **`smiles`** list, to which the addition operation is applied using a second, randomly selected fragment from the same list. Further application of the addition operation with further fragments is carried out until a randomly selected size within the limits given by **`molsize`** is reached. A large number (500 in this work) of mutation operations using the **`smarts`** variable are then applied to the molecule.

New generations of molecules are created using a elitism rate of 10% so that the new population is made from the top 10% best performing molecules from the previous population. The remaining 90% is made using child molecules created with the following procedure. Two 2-way tournament selections are carried out where in each tournament a molecule is selected out of a set of two randomly selected molecules from the previous population with a probability of 75% to select the fitter molecule. The two molecules from the two 2-way tournaments are then used to create two child molecules using the **`Crossover`** transformation. Each child molecule then has a probability of

5% to undergo **Mutation** and a probability of 5% to undergo **Recombination**.

The search algorithm therefore runs by creating a initial population where in this report we have used a population size of 100. The fitness of each molecule in the initial population is evaluated and the next generation is created from this. Newer generations are created continually until a desired number of generations or a convergence criteria is reached. In this report we have chosen to run all searches for a total of 30 generation. Since the selection and replication for the creation of new molecules in the next generation favour fitter molecules the search algorithm is therefore driven to a global minimum or maximum.

S2 Full details of the chemical search space

In this report we have chosen to explore the region of chemical space of aza-pentacene type molecules. To carried this out with algorithm described in Section S1 requires the input variables written in Listing 1. We were able to determined the size of the chemical space that can be explored to contain 68064 unique molecules which was obtained by running the randomised molecule creation in a infinite loop and converting each molecule generated into its canonical InChi string[4] and was then added into a Python set. The program was left to run until no further changes to the number of elements in the set had occurred over a few days.

```
smiles = ['c1ccccc1', 'c1c**cc1']
smarts = ['[#6R1&H]', '[#7R1&H0]']
molsize = [5, 5]
```

Listing 1: Input variables used in this report which define a chemical space of aza-pentacene type molecules to be searched.

S3 Fitness functions

In this report we have designed two different fitness functions,

$$F_A = \lambda_- \tag{1}$$

$$F_B = \lambda_- + \Phi \qquad \Phi = \begin{cases} W - \mathrm{A_s} & \mathrm{A_s} < W \\ 0 & \mathrm{A_s} \geq W \end{cases} \tag{2}$$

where λ_- is the reorganisation energy for electron transport between two molecules approximated using the four-point scheme using isolated molecule energies.[5]

$$\lambda_- = \Big[E_-(R_0) - E_0(R_0)\Big] + \Big[E_0(R_-) - E_-(R_-)\Big] \tag{3}$$

The penalty function where $\Phi = W - \mathrm{A_s}$ is the equation for the Schottky barrier from the Schottky-Mott rule for the injection of an electron from an electrode with a work function W into the semiconductor material with a the solid-state electron affinity $\mathrm{A_s}$.[6, 7] Where the penalty is only applied for cases below the target work function to favour less reactive higher work function metals. For this report we will be using $W = 4.1$ eV which we have chosen to match more closely the work function of metals such as Ag, Cu and Au which have the values 4.26, 4.65, and 5.1 eV.

The fitness function F_A was used to search for molecules with the best probabilities to produce crystal structures with high electron mobilities when using the transport hopping models. Additionally we use F_A to evaluate the performance and reproducibility of the evolutionary algorithm since the global minimum was expected to correspond to pentacene as any aza-substitution or non-linearity is expected to disrupt delocalisation of the excess electron and increase its reorganisation energy. The fitness function F_B includes the additional penalty function to ensure larger electron affinities and therefore a smaller Schottky barrier when using higher work function electrodes in OFETs. Therefore F_B is used to minimise both the barrier for injection of an electron into the semiconductor and the barrier for hopping across the semiconductor in hopping transport models.

Both fitness functions were evaluated by taking each molecule generated by the evolutionary algorithm and creating 3D molecular geometries using the RDKit inbuilt initial coordinates generation and UFF optimisation functions.[8] The UFF geometries are then taken for a further optimisation step carried out at the B3LYP/6–311+G** level of theory to generate the neutral geometries and then used to determine the geometries of the charged species. Solid-state electron affinities were approximated from gas phase adiabatic electron affinities which were calculated using energies extracted from the the geometry optimisation calculations. Electron reorganisation energies are obtained by carrying out two additional single point energy calculation at the same level of theory where all DFT calculations for the fitness evaluation were carried out using GAUSSIAN09.[9]

Solid-state electron affinities were approximated from calculated gas phase adiabatic electron affinities by taking advantage of the known correlation between the two values.[10, 11] Here we calculated the gas phase electron affinities for 12 molecules to produced a linear regression fit against experimental low-energy inverse photoemission spectroscopy (LEIPS) values for thin-films organic semiconductors with constants ($m = 1.00$, $c = 1.11$) and an $R^2 = 0.97$, see Section S4. Therefore solid-state electron affinity can be obtained from gas phase calculations by making a very simple correction of 1.1 eV to the gas phase adiabatic electron affinities calculations.

$$\mathrm{A_s} \approx \Big[E_0(R_0) - E_-(R_-)\Big] + 1.1 \tag{4}$$

Using this simple adjustment with $W = 4.1$ eV will mean the fitness function F_B will also be

equivalent applying a penalty function similar to Φ against molecules with gas phase electron affinities greater then 3.0 eV.

S4 Fitting DFT isolated molecule electron affinities to averaged solid-state low-energy inverse photoemission spectroscopy (LEIPS) electron affinities

Initial starting geometries for the molecules 4CzIPN, Alq3, BCP, CBP, Liq and PCBM were obtained by extracting coordinates from crystal structures KANYUU, TICBUD, TUGDOV, ADATOP, QATMON and PESJII01 respectively to ensure the correct conformers were used during DFT electron affinity calculations. Initial starting geometries for the remaining molecules C60, C70, PEN, PFP, PNQ and PTCDA were obtained using inbuilt RDKit initial coordinates generation and UFF optimisation function as these molecules are π-bonded rigid molecules that are unlikely to have more than one conformer. Adiabatic electron affinities were calculated by carrying out an geometry optimisations using B3LYP/6–311+G** at the neutral followed by another geometry optimisation for the charged state. Adiabatic electron affinities are there calculated from the differences in the SCF energies between the two states.

Experimental thin-film solid-state electron affinities can vary depending the orientation of the molecules that are deposited on the substrate. For example pentacene takes on a standing orientation on a SiO_2 substrate with an experimental LEIPS electron affinity of 2.35 eV and a lying orientation on the highly oriented pyrolytic graphite (HOPG) substrate with a electron affinity of 3.14 eV.[12] Since we are optimising for only the molecular structure we average solid-states electron affinity of all known experimental values to fit against DFT results to estimate what the typical Schottky barrier for the crystal structures that a molecules might form. Experimental values for the solid-state electron affinities and DFT calculated gas phase electron affinities are shown in Table S1. The average experimental and DFT calculated values are plotted with a linear regression model in Fig. S3.

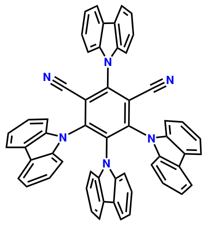

4CzIPN

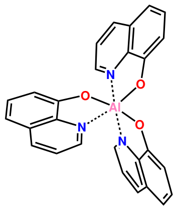

Alq_3

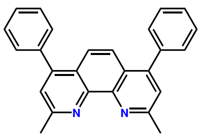

BCP

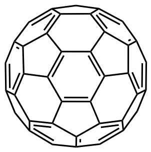

C60

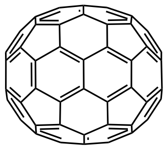

C70

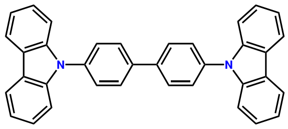

CBP

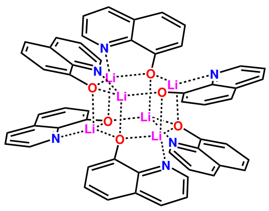

Liq

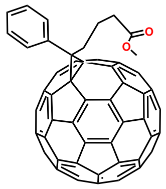

PBCM

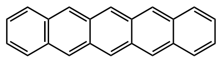

PEN

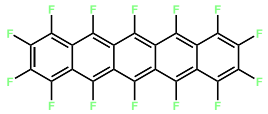

PFP

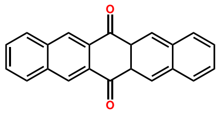

PNQ

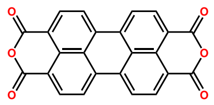

PTCDA

Figure S2: Chemical diagrams of the molecules used to fit calculated gas phase electron affinities to experimental solid-state electron affinities.

Molecule	DFT A_g	LEIPS $\bar{A}_s$	LEIPS A_s	References
4CzIPN	2.03	2.81	2.81	[11]
Alq_3	1.02	2.06	2.06	[11]
BCP	0.616	1.89	1.89	[11]
C60	2.64	3.98	3.98	[13]
C70	2.69	4.00	4.00	[13]
CBP	0.786	1.75	1.75	[11]
Liq	0.749	1.85	1.85	[11]
PCBM	2.53	3.75	3.64, 3.76, 3.84	[13, 14]
PEN	1.54	2.73	2.35, 2.70, 3.14	[12, 15]
PFP	2.85	3.85	3.58, 4.12	[12]
PNQ	1.65	2.83	2.34, 2.83, 3.32	[16]
PTCDA	3.17	4.11	4.11	[17]

Table S1: A table showing the calculated B3LYP/6–311+G** gas phase adiabatic electron affinities, averaged experimental LEIPS solid-state electron affinities and experimental LEIPS with solid-state electron affinities with different molecular orientations of the thin film on the OFET substrate.

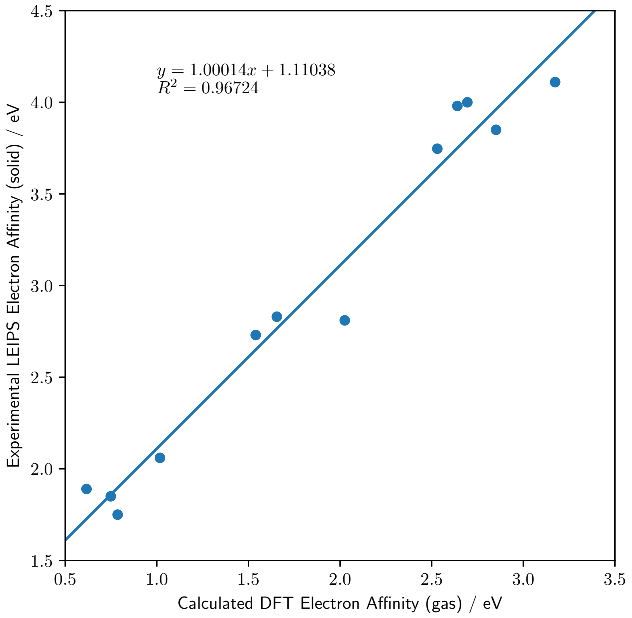

Figure S3: Linear fit between the averaged experimental LEIPS solid-state electron affinities and calculated B3LYP/6–311+G** gas phase adiabatic electron affinities.

S5 Comparison of calculated mobilties between Marcus theory and non-adiabatic molecular dynamics for a set of tetracene systems.

We carried out comparisons of calculated mobilities using Marcus theory, as outlined in our paper, against non-adiabatic molecular dynamics simulations.[18–21] This method has been shown to obtain excellent correlation with experimental mobilities[22] which therefore will be a good reference to compare against. For the test set, we use a series of substituted tetracene systems whose hole mobilities were recently evaluated.[23]

We started with the experimentally determined crystal structures of each crystal structure. Hydrogen bond lengths are typically underestimated in experimental crystal structures, and an equilibration step is carried out in the non-adiabatic molecular dynamics simulations. Therefore, we give a fair comparison to our method by taking molecular geometries from the experimental crystal structures and reoptimise them using B3LYP/6-311G** with heavy atom positions fixed so that the correct conformations are maintained. These molecular geometries are then pasted back into the experimental crystal structures which are then optimised using the lattice energy minimisation method as outlined in Section S7. Mobilities for these optimised structures are then obtained by running kinetic Monte Carlo simulations using Marcus theory rates as outlined in the paper. Results are shown in Table S2 and Fig. S4.

System	Database Identifier	μ / cm^2(Vs)$^{-1}$ (Marcus Theory)	μ / cm^2(Vs)$^{-1}$ (Non-adiabatic Molecular Dynamics)	Electronic Coupling (Non-adiabatic Molecular Dynamics)
TET	GUMZOE	0.32	0.6	18.4 ± 33.6
TiPeT	DUWHOT	0.68	2.7	76.2 ± 29.4
TETCEN	TETCEN	3.19	3.5	88.7 ± 32.4
TMT	GUMZIY	3.54	20.8	146.1 ± 21.8
DPrT	DUWPER	8.62	8.2	101.5 ± 27.3
TPeT	GUNBAT	10.84	9.6	122.4 ± 25.7
TPrT	GUMZUK	11.11	15.8	143.4 ± 23.3
TiBuT	DUWHEJ	12.86	1.3	124.1 ± 56.3
TBuT	HIGNIV	14.25	13.2	156.2 ± 25.2
THT	GUNBEX	14.32	11.0	137.1 ± 28.3

Table S2: Table showing calculated mobilities calculated using kinetic Monte Carlo using Marcus theory rates and non-adiabatic molecular dynamics simulations for 10 different tetracene type systems and the largest electronic coupling with deviations from the non-adiabatic molecular dynamics simulations.[23] TMT, TET, TPrT, TBuT, TPeT, and THT are tetra- methyl-, ethyl-, propyl-, butyl-, pentyl-, and hexyl-tetracene, respectively. TiBuT and TiPeT are iso-butyl and iso-pentyl tetracene. TPrT is 1,4-propyl tetracene. TETCEN is tetracene.

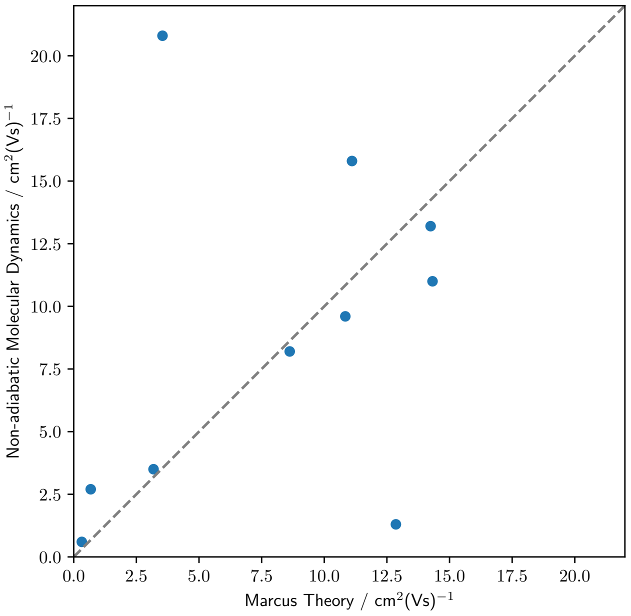

Figure S4: Scatter plot of hole mobilities from kinetic Monte Carlo simulations using Marcus theory rates against hole mobilities from non-adiabatic molecular dynamics simulations, $y = x$ is indicated by the dashed line.

In general, we see fairly good ranking for these systems with small/large mobilities predicted by Marcus theory corresponding to small/large mobilities with non-adiabatic molecular dynamics. There are two outliers: the systems TiBuT and TMT. For TiBuT, the hole mobility predicted by Marcus theory is dramatically overestimated. This could be due to the large variation in its electronic coupling ±56.3 eV through the molecular dynamics, indicating an important effect of phonons on hole transport. The hole mobility in TMT, with a smaller deviation in its electronic coupling of ±21.8 eV, is underestimated. The mobility in TMT from non-adiabatic molecular dynamics relies on a complex pathway, where the mobility may depend strongly on dynamics connecting 'columns' of molecules.[23] These errors are probably acceptable for our use case since

each molecule may be predicted to include up to a hundred or more crystal structures in its low energy region and the likelihoods of a molecule to produce a high performance organic semiconductor is assessed using a weighted average and deviation over its crystal structure landscape.

In future work it will however be favourable to replace Marcus theory with models which include effects due to the non-local coupling, a parameter which causes the deviation in the electronic coupling. These effects are taken into account in, for example, the transient localisation theory.[24–26] However these methods incur an increased computational expense and future work is required to see if they can be used as a part of a high throughput screening program. This not only requires that these methods are computationally inexpensive but that they are easily automated and require no human intervention at any point. There are therefore significant challenges to replacing Marcus theory especially since it will be necessary to scale up the material discovery program we have developed here by at least an order of magnitude.

S6 Definition of molecular non-linearity

We define the amount of non-linearity by the number of bonds that connect two rings together that are not the intersecting bonds.

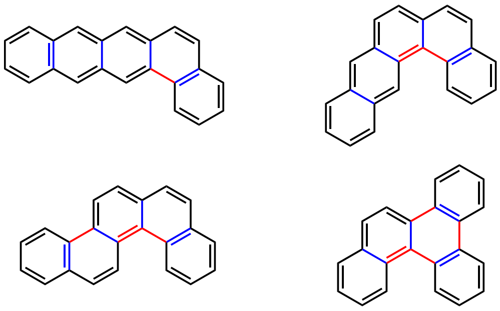

Figure S5: Example chemical structures with increasing non-linearity from TOP left to bottom right. Where we have define the amount of non-linearity by the number of bonds (red) that connect two rings together that are not the intersecting bonds (blue).

S7 Full details of crystal structure prediction calculations

Crystal structure prediction calculations were performed for the most promising molecules identified from the evolutionary search, using the Global Lattice Energy Explorer (GLEE) program.[27] A low-discrepancy, quasi-random sampling of crystal packing variables was used to uniformly sample the lattice energy surface of each molecule. Trial crystal structures were generated and lattice energy minimised until a total of 34,000 successfully lattice energy minimised crystal structures were produced for each molecule in the most commonly observed space groups for organic molecules: 4000 structures in each of ($P2_1/c$, $P\bar{1}$, $C2/c$, $P2_12_12_1$, $P2_1$, $Pbca$) and 2000 in each of ($P1$, $C2$, Cc,

$Pna2_1$, $Pca2_1$) with one molecule in the asymmetric unit ($Z' = 1$).

Lattice energies were calculated with the W99 intermolecular atom-atom potential[28] combined with an atomic multipole electrostatic model based on the molecular charge densities calculated from a distributed multipole analysis[29] of the B3LYP/6–311G** density, with multipoles up to hexadecapole on each atom. Ewald summation was used for charge-charge, charge-dipole and dipole-dipole interactions, while all higher order electrostatics and repulsion-dispersion interactions were calculated to a 35 Å cutoff. All lattice energy minimisations were performed using the DMACRYS software,[30] with space group symmetry constrained throughout the optimisations. Molecular geometries were kept rigid, constrained at their neutral isolated molecule optimised geometries using the B3LYP/6–311G** level of theory. The removal of duplicate crystal structures from the final structure sets was performed across all space groups by comparing predicted X-ray diffraction patterns calculated using PLATON.[31]

S8 Energy-Structure-Function maps of electron mobility in the optimised molecules

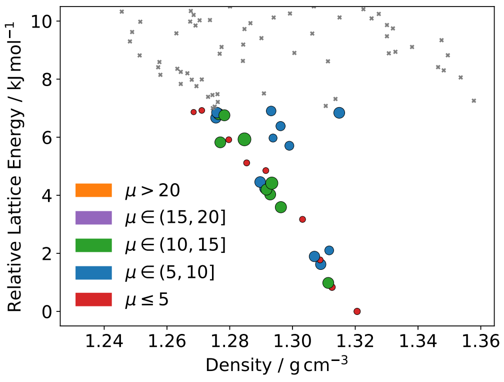

1A

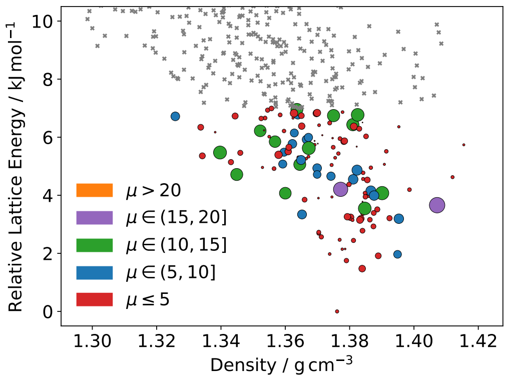

2A

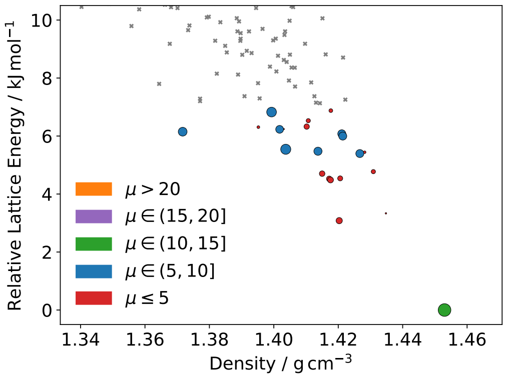

3A

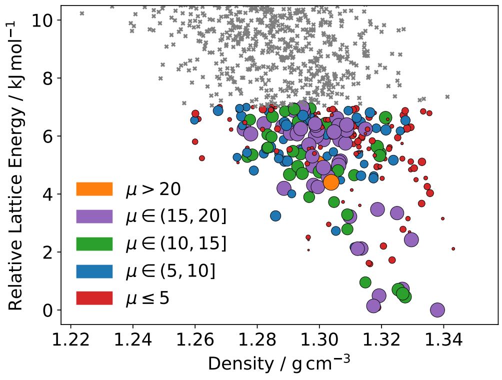

4A

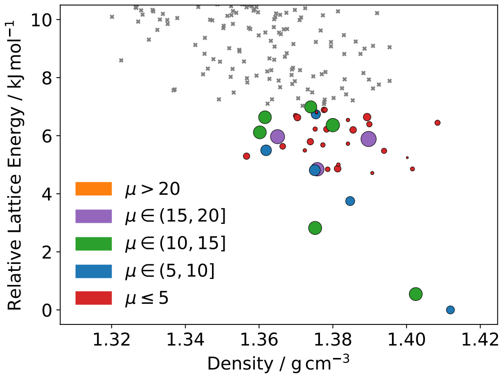

5A

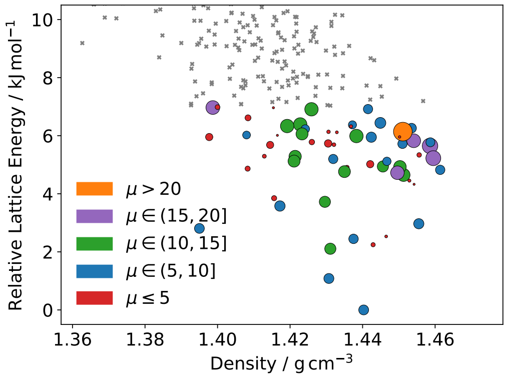

6A

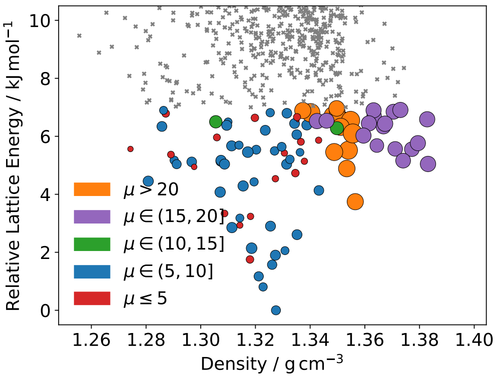

7A

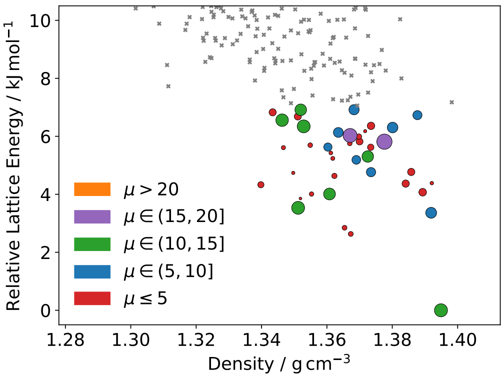

8A

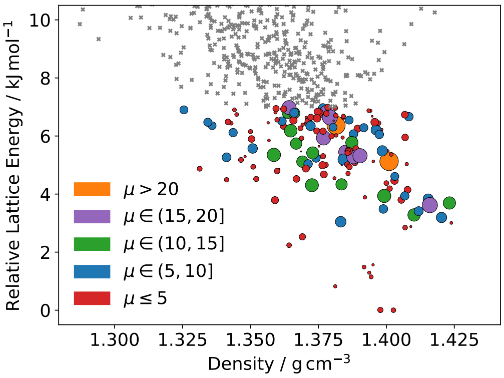

9A

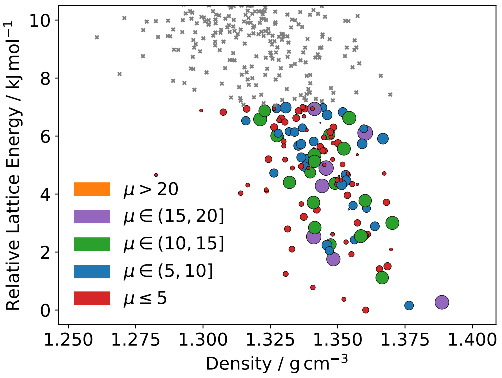

10A

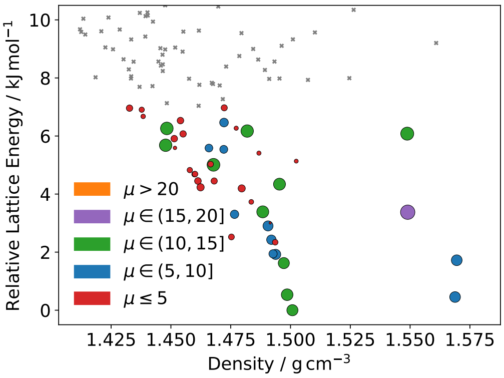

1B

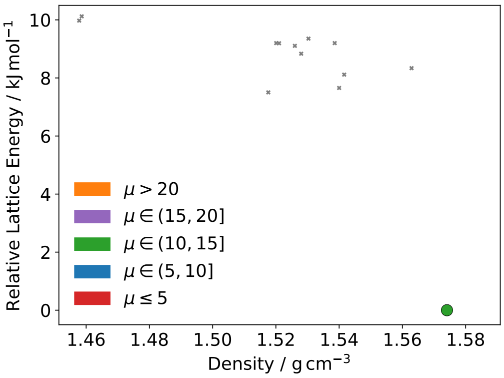

2B

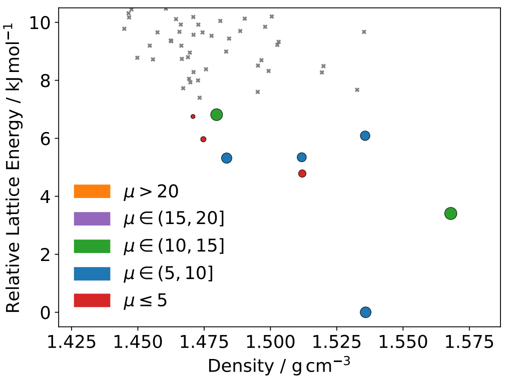

3B

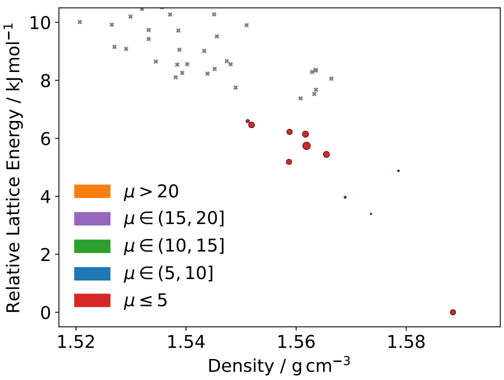

4B

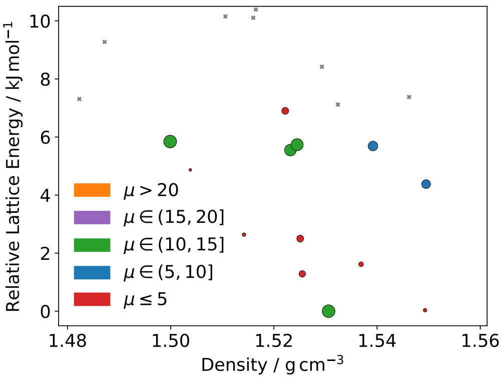

5B

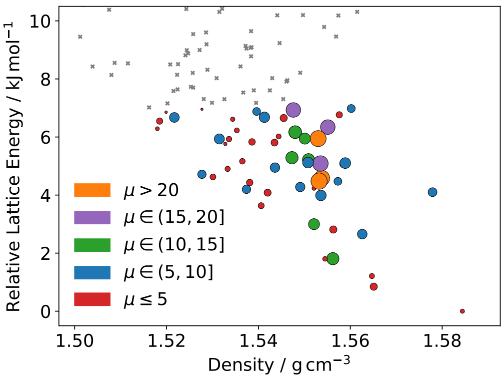

6B

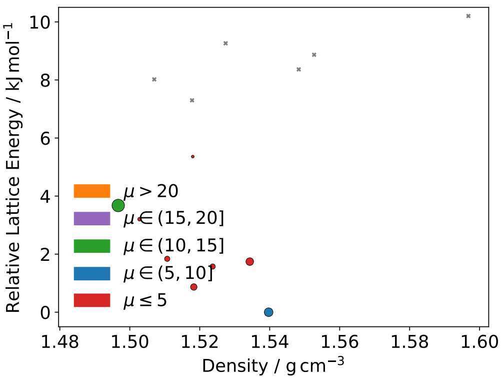

7B

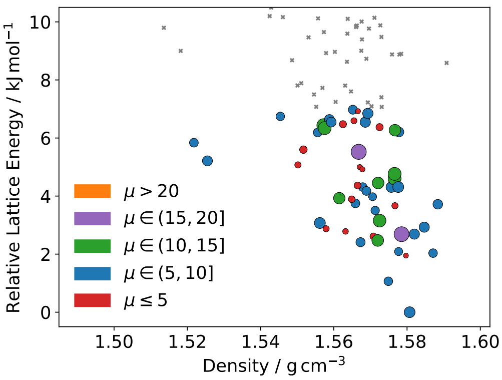

8B

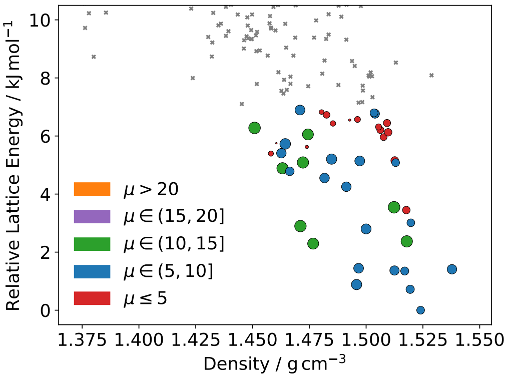

9B

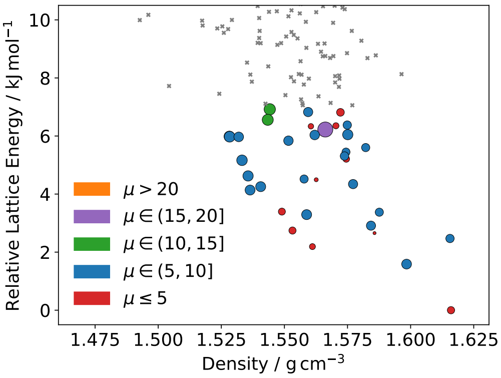

10B

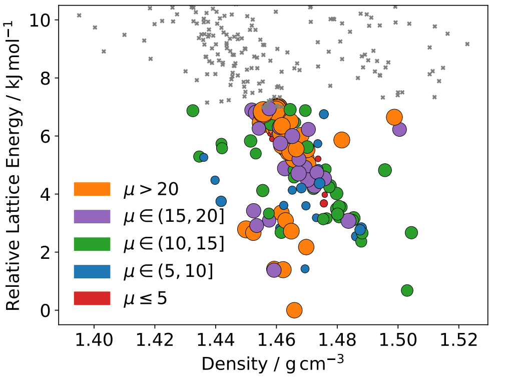

WH5A

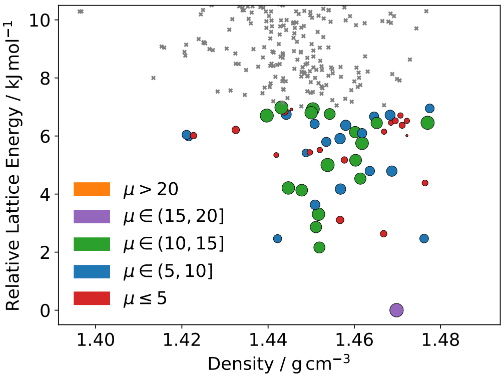

WH5B

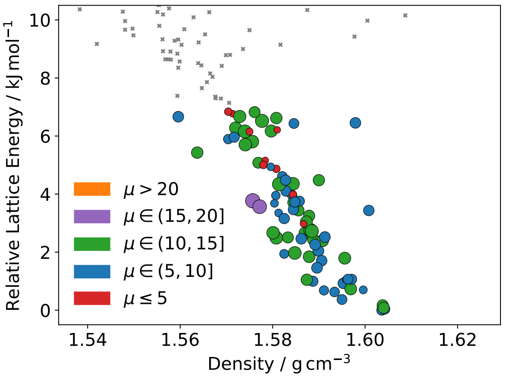

WH7A

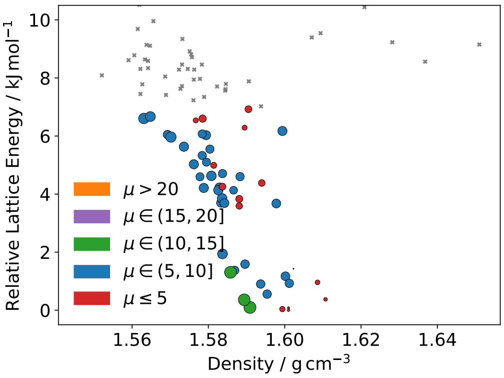

WH7B

References

[1] D. Weininger, *Journal of Chemical Information and Computer Sciences*, 1988, **28**, 31–36.

[2] D. Weininger, A. Weininger and J. L. Weininger, *Journal of Chemical Information and Computer Sciences*, 1989, **29**, 97–101.

[3] R. Sayle, *1st-class SMARTS patterns*, 1997.

[4] S. R. Heller, A. McNaught, I. Pletnev, S. Stein and D. Tchekhovskoi, *Journal of Cheminformatics*, 2015, **7**, 23.

[5] H. Oberhofer, K. Reuter and J. Blumberger, *Chem. Rev.*, 2017, **117**, 10319.

[6] R. T. Tung, *Applied Physics Reviews*, 2014, **1**, 011304.

[7] M. Waldrip, O. D. Jurchescu, D. J. Gundlach and E. G. Bittle, *Advanced Functional Materials*, 2019, 1904576.

[8] G. Landrum, *RDKit: Open-source cheminformatics*, `http://www.rdkit.org`.

[9] M. J. Frisch, G. W. Trucks, H. B. Schlegel, G. E. Scuseria, M. A. Robb, J. R. Cheeseman, G. Scalmani, V. Barone, B. Mennucci, G. A. Petersson, H. Nakatsuji, M. Caricato, X. Li, H. P. Hratchian, A. F. Izmaylov, J. Bloino, G. Zheng, J. L. Sonnenberg, M. Hada, M. Ehara, K. Toyota, R. Fukuda, J. Hasegawa, M. Ishida, T. Nakajima, Y. Honda, O. Kitao, H. Nakai, T. Vreven, J. A. Montgomery, Jr., J. E. Peralta, F. Ogliaro, M. Bearpark, J. J. Heyd, E. Brothers, K. N. Kudin, V. N. Staroverov, R. Kobayashi, J. Normand, K. Raghavachari, A. Rendell, J. C. Burant, S. S. Iyengar, J. Tomasi, M. Cossi, N. Rega, J. M. Millam, M. Klene, J. E. Knox, J. B. Cross, V. Bakken, C. Adamo, J. Jaramillo, R. Gomperts, R. E. Stratmann, O. Yazyev, A. J. Austin, R. Cammi, C. Pomelli, J. W. Ochterski, R. L. Martin, K. Morokuma, V. G. Zakrzewski, G. A. Voth, P. Salvador, J. J. Dannenberg, S. Dapprich, A. D. Daniels, O. Farkas, J. B. Foresman, J. V. Ortiz, J. Cioslowski and D. J. Fox, *Gaussian 09*, 2009.

[10] P. Schwenn, P. Burn and B. Powell, *Org. Electron.*, 2011, **12**, 394 – 403.

[11] H. Yoshida and K. Yoshizaki, *Org. Electron.*, 2015, **20**, 24 – 30.

[12] H. Yoshida, K. Yamada, J. Tsutsumi and N. Sato, *Phys. Rev. B*, 2015, **92**, 075145.

[13] H. Yoshida, *The Journal of Physical Chemistry C*, 2014, **118**, 24377–24382.

[14] Y. Zhong, S. Izawa, K. Hashimoto, K. Tajima, T. Koganezawa and H. Yoshida, *The Journal of Physical Chemistry C*, 2015, **119**, 23–28.

[15] W. Han, H. Yoshida, N. Ueno and S. Kera, *Applied Physics Letters*, 2013, **103**, 123303.

[16] K. Yamada, S. Yanagisawa, T. Koganezawa, K. Mase, N. Sato and H. Yoshida, *Phys. Rev. B*, 2018, **97**, 245206.

[17] H. Yoshida, *Journal of Electron Spectroscopy and Related Phenomena*, 2015, **204**, 116 – 124.

[18] J. Spencer, F. Gajdos and J. Blumberger, *The Journal of Chemical Physics*, 2016, **145**, 064102.

[19] A. Carof, S. Giannini and J. Blumberger, *The Journal of Chemical Physics*, 2017, **147**, 214113.

[20] S. Giannini, A. Carof and J. Blumberger, *The Journal of Physical Chemistry Letters*, 2018, **9**, 3116–3123.

[21] A. Carof, S. Giannini and J. Blumberger, *Phys. Chem. Chem. Phys.*, 2019, **21**, 26368–26386.

[22] S. Giannini, A. Carof, M. Ellis, H. Yang, O. G. Ziogos, S. Ghosh and J. Blumberger, *Nature Communications*, 2019, **10**, 3843.

[23] O. G. Ziogos, S. Giannini, M. Ellis and J. Blumberger, *J. Mater. Chem. C*, 2020, **8**, 1054–1064.

[24] S. Ciuchi, S. Fratini and D. Mayou, *Phys. Rev. B*, 2011, **83**, 081202.

[25] S. Fratini, D. Mayou and S. Ciuchi, *Advanced Functional Materials*, 2016, **26**, 2292–2315.

[26] T. Nematiaram, S. Ciuchi, X. Xie, S. Fratini and A. Troisi, *The Journal of Physical Chemistry C*, 2019, **123**, 6989–6997.

[27] D. H. Case, J. E. Campbell, P. J. Bygrave and G. M. Day, *J. Chem. Theory Comput.*, 2016, **12**, 910–924.

[28] E. W. Donald, *Journal of Computational Chemistry*, 2001, **22**, year.

[29] A. Stone and M. Alderton, *Molecular Physics*, 2002, **100**, 221–233.

[30] S. L. Price, M. Leslie, G. W. A. Welch, M. Habgood, L. S. Price, P. G. Karamertzanis and G. M. Day, *Phys. Chem. Chem. Phys.*, 2010, **12**, 8478–8490.

[31] A. L. Spek, *Acta Cryst. D*, 2009, **65**, 148–155.
